# Tailored UPRE2 variants for dynamic gene regulation in yeast

**DOI:** 10.1073/pnas.2315729121

**Published:** 2024-04-30

**Authors:** Chufan Xiao, Xiufang Liu, Yuyang Pan, Yanling Li, Ling Qin, Zhibo Yan, Yunzi Feng, Mouming Zhao, Mingtao Huang

**Affiliations:** ^a^School of Food Science and Engineering, South China University of Technology, Guangzhou 510641, China

**Keywords:** UPRE2 variants, Hac1, unfolded protein response, responsive promoter

## Abstract

Genetic elements are foundational in synthetic biology serving as vital building blocks. They enable programming host cells for efficient production of valuable chemicals and recombinant proteins. The unfolded protein response (UPR) is a stress pathway in which the transcription factor Hac1 interacts with the upstream unfolded protein response element (UPRE) of the promoter to restore endoplasmic reticulum (ER) homeostasis. Here, we created a UPRE2 mutant (UPRE2m) library. Several rounds of screening identified many elements with enhanced responsiveness and a wider dynamic range. The most active element m84 displayed a response activity 3.72 times higher than the native UPRE2. These potent elements are versatile and compatible with various promoters. Overexpression of *HAC1* enhanced stress signal transduction, expanding the signal output range of UPRE2m. Through molecular modeling and site-directed mutagenesis, we pinpointed the DNA-binding residue Lys60 in Hac1(Hac1-K60). We also confirmed that UPRE2m exhibited a higher binding affinity to Hac1. This shed light on the mechanism underlying the Hac1-UPRE2m interaction. Importantly, applying UPRE2m for target gene regulation effectively increased both recombinant protein production and natural product synthesis. These genetic elements provide valuable tools for dynamically regulating gene expression in yeast cell factories.

## Significance

The optimization of gene expression in yeast cell factories is pivotal for maximizing product production. The unfolded protein response (UPR) pathway, through its specific elements, offers a route to achieving this goal. Our study develops additional UPRE2 variants, surpassing their native counterparts in responsiveness and dynamic range, streamlining the engineering of yeast strains. By pinpointing key interactions, like Hac1-K60, and demonstrating the increased binding affinity between UPRE2 mutant (UPRE2m) and Hac1, we deepen our understanding of the molecular mechanism that governs these pathways. These findings not only elevate the precision with which we can harness yeast cells for bioproduction but also represent a significant progress in our toolkit for synthetic biology, emphasizing its potential in cell factory design and construction.

The yeast *Saccharomyces cerevisiae* is frequently utilized as a production system for recombinant proteins (1, 2). The endoplasmic reticulum (ER) is pivotal in many cellular functions, including secretory and membrane protein folding, calcium homeostasis, and lipid biosynthesis (3–5). Exceeding the protein production capacity of yeast can lead to misfolded protein generation in the ER. The accumulation of these misfolded proteins stresses the cell and triggers the cellular stress response pathway. The unfolded protein response (UPR) pathway, an adaptive signaling mechanism, maintains ER homeostasis (6). Within this network, the ER transmembrane sensor molecule, Ire1, mediates signal transduction by excising a 252-nucleotide intron from the precursor *HAC1* mRNA, which then gets translated into Hac1. This transcription factor, Hac1, interacts with the unfolded protein response element (UPRE) promoter region, enhancing protein folding capacity by facilitating the transcription of associated genes (7). However, if ER stress becomes excessive or prolonged, the UPR might not mitigate accumulated misfolded proteins, potentially leading to cell death (8). Recent studies in animals and humans indicate the significant roles of ER stress and the UPR in immune responses, cardiovascular diseases, and Alzheimer’s disease (9, 10).

Fluorescent proteins under the control of UPRE-mediated promoters can serve as UPR biosensors to detect ER stress caused by the accumulation of unfolded proteins (11). Such biosensors provide a quick method to evaluate the secretory capacity of strains, thereby indicating the effectiveness of the strategies employed in strain engineering for protein production (12). Utilizing UPR-responsive promoters in the metabolic engineering of yeast strains has proven beneficial for optimizing gene expression and enhancing protein production. This strategy balances gene expression with cellular resource requirements during the production phase (13, 14). Currently, UPR-based transcriptional sensors mainly utilize a limited combination of UPRE with native non-UPR promoters, like *CYC1*p and *TDH3*p, or are restricted to a selected group of natural UPR promoters, such as *HAC1*p, *KAR2*p, and *ERO1*p (15, 16). UPRE1 serves as the cis-acting element associated with the *KAR2* promoter, regulating its activity (17). UPRE2 was first identified upstream of the *ERO1* promoter (18). Compared with UPRE1, UPRE2 exhibits a broader dynamic range and an enhanced stress response for promoter (13). The interaction between UPRE2 and Hac1 has been examined in vitro using systematically designed DNA fragments. This study revealed that UPRE2 recognition requires a region homologous to the N-terminal extension of the primary DNA-binding domain (19). Nevertheless, the distinct residues within the extended region influencing target DNA affinity remain to be elucidated.

**Fig. 1.**
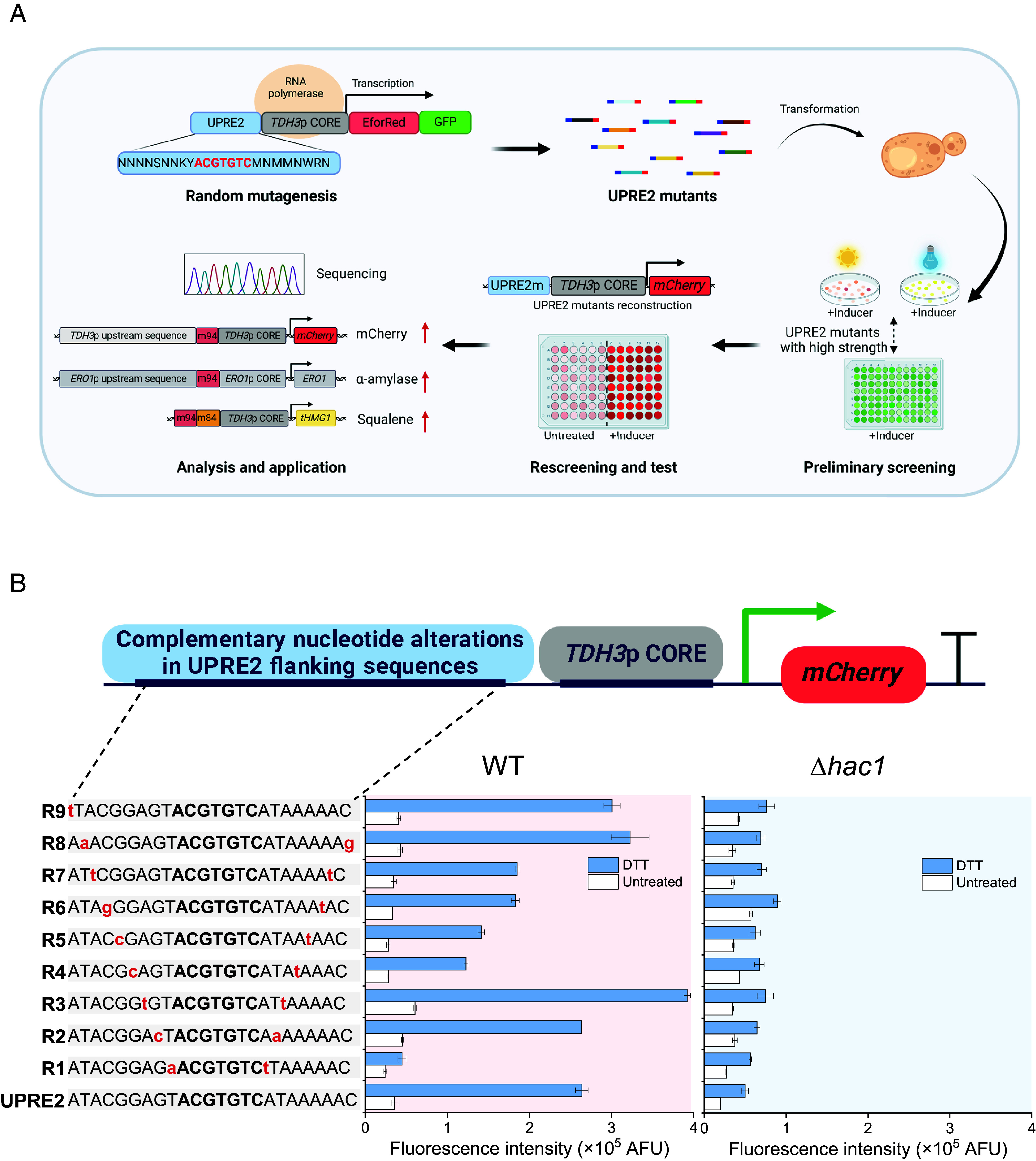
UPRE2 flanking sequences influence stress responses. (*A*) Schematic diagram of UPRE2m library creation and screening (created with BioRender.com). (*B*) Response of hybrid promoter *TDH3*p CORE with the complementary nucleotide alterations in UPRE2 flanking sequences to 5 mM DTT treatment in WT and Δ*hac1* strains. Data shown are mean values ± SDs of biological duplicates of single clones.

To address constraints concerning the variety and responsiveness of UPRE2 elements, we created a library of UPRE2 mutants (UPRE2m), from which the optimal candidates were discerned. Additionally, by examining the behavior of UPRE2m in Hac1 mutants, we pinpointed the essential protein residues for UPRE2m target site recognition. These engineered UPRE2m can be inserted upstream of a promoter for cell state monitoring or for regulating metabolic node genes (Fig. 1*A*). Our study created responsive elements with enhanced capabilities and shed light on the fundamental mechanisms through which Hac1 recognizes target genes.

## Results and Discussion

### Construction of the UPRE2 Variant Library.

The core sequence of UPRE2, 5′-ACGTGTC-3′, is fundamental for its function. While the core sequence is well understood, the modularity of its adjacent sequences remains elusive (19, 20). Introducing point mutations into sequences adjacent to the UPRE2 core markedly affected gene expression (21). UPRE2 sequences from the known Hac1 target promoters, such as *ERO1*p, *KAR2*p, *SIL1*p, and *HNT1*p (22, 23), and those *ERO1*p homologous promoters regulated by Hac1 from different species, show consistent nucleotide conservation surrounding the core sequence (*SI Appendix*, Fig. S1). To evaluate the influence of these adjacent sequences, we systematically truncated the flanking sequences of the full-length UPRE2 and also created complementary nucleotide alterations in flanking sequences, while maintaining the integrity of the core sequence 5′-ACGTGTC-3′. We then examined the response of the hybrid promoter *TDH3*p CORE, integrated with either the truncated UPRE2 or UPRE2 with complementary nucleotide alterations in its flanking sequences, in both wild-type (WT) and *Δhac1* strains. Dithiothreitol (DTT) is a commonly used inducer of ER stress by inhibiting disulfide bond formation (24). Upon DTT treatment, hybrid promoters with the truncated UPRE2 sequence showed varying response activities in the WT strain. Among the hybrid promoters with complementary nucleotide alterations, R1, R4, R5, R6, and R7 exhibited decreased activity in the WT strain. Conversely, hybrid promoters R3, R8, and R9 showed increased activity in the WT strain. However, the response activity of all hybrid promoters was significantly reduced in the *Δhac1* strain (Fig. 1*B* and *SI Appendix*, Fig. S1*D*). These findings underscore the importance of the UPRE2 flanking region in the transcription function of the core sequence.

To diversify UPRE2 elements, mutations were introduced into the flanking sequences of UPRE2 using PCR-based techniques. The fusion protein EforRed-GFP was employed as a reporter for rapid UPRE2m screening (*SI Appendix*, Fig. S2*A*). EforRed, a chromoprotein, has a strong absorption of visible light, making its color perceptible even in daylight (25). This chromoprotein was used for preliminary screening through agar plate coloration, and GFP was used for quantification with a plate reader (Fig. 1*A*). To ascertain the optimal intensity of element expression, promoters of varying strengths (*TDH3*p > *TEF1*p > *TPI1*p > *CYC1*p) (26) were selected to express the fusion protein on high-copy and low-copy plasmids, and also directly on chromosomes (*SI Appendix*, Fig. S2*B*). As the culture time increased, the accumulation of fusion protein resulted in progressive deepening of its color under blue light exposure. While expression of the fusion protein on the high-copy plasmid p426 generated a noticeable background noise, there was distinct correlation between promoter strength and colony color depth when expressed on the low-copy plasmid p416 or on the chromosome (*SI Appendix*, Fig. S2*C*). The fluorescence intensity of GFP aligned with the promoter strength, indicating the reliability of the fusion protein as a reporter for subsequent screening (*SI Appendix*, Fig. S2*D*). Based on these findings, a library of 10^5^ UPRE2 variants was constructed by integrating expression cassettes into the chromosome (*SI Appendix*, Fig. S2*E*).

### Characterization of the UPRE2m Library.

**Fig. 2.**
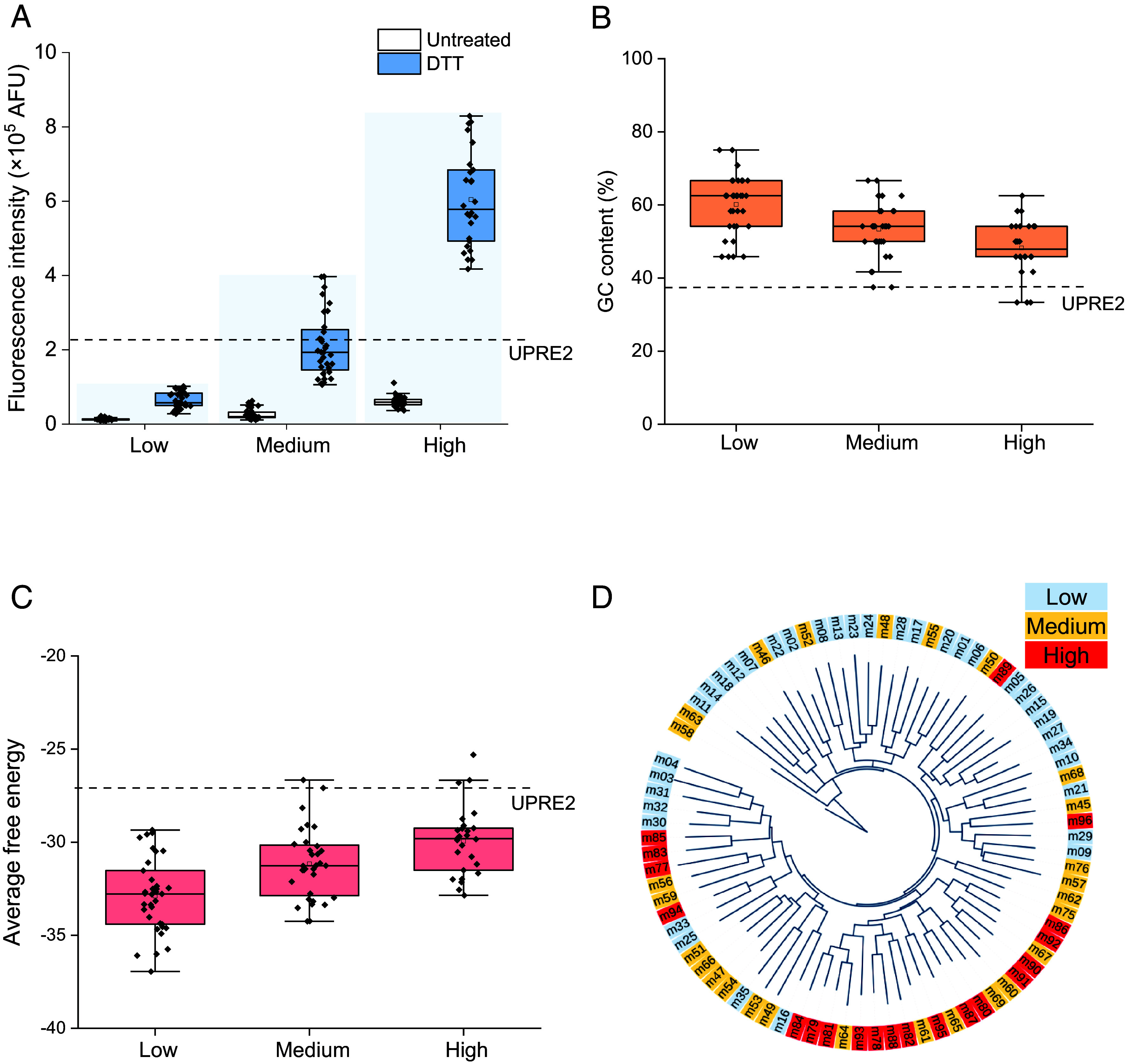
Analysis of UPRE2m sequences. (*A*) Response of hybrid promoter UPRE2m-*TDH3*p CORE upon 5 mM DTT treatment. (*B*) GC content in different UPRE2m groups. The dashed line indicates GC content of the native UPRE2. (*C*) Free energy of UPRE2m in different groups. In the box plots, whisker lengths correspond to the dataset’s minimum and maximum values. The upper and lower whiskers extend to the maximum and minimum values, respectively. The lines within the box plot indicate the median and quartiles. (*D*) Phylogenetic analysis of UPRE2m.

In the preliminary screening, approximately 400 strains that exhibited higher light intensity underwent secondary validation. The UPRE2m sequence was then amplified from these strains and positioned upstream of *TDH3*p CORE, using mCherry as the reporter. The reconstituted UPRE2 mutants were subsequently tested for their response in both untreated and DTT-treated conditions in well plates (Fig. 1*A*). Using the response intensity of the standard hybrid promoter UPRE2–*TDH3*p CORE as a benchmark (~2.5 × 10^5^ AFU), approximately 100 mutants were chosen and divided into three groups (~30 mutants in each group): low (<1 × 10^5^ AFU); medium (1 × 10^5^ ~ 4 × 10^5^ AFU); and high (>4 × 10^5^ AFU). These mutants underwent sequencing to determine their sequence composition (*SI Appendix*, Table S1). Among them, the mutant with the highest response activity showed an activity approximately 3.72 times greater than the standard UPRE2. Notably, there was a 29-fold activity difference between the most and least responsive mutants (Fig. 2*A*). Furthermore, UPRE2m-*TDH3*p CORE responses with varying activities showed similar trends at different time points (*SI Appendix*, Fig. S3).

DNA molecules exhibit inherent polymorphism, with their structures being influenced by both base sequences and the surrounding environment (27). Promoter regions exhibit unique structural properties, including low stability and less bendability. The responsive expression of the gene is closely related to the structural characteristics of DNA and the architecture of the promoter region (28, 29). Notably, the high responsive activity group of UPRE2m has lower GC content and higher free energy content (Fig. 2 *B* and *C*). Phylogenetic analysis of UPRE2m sequences revealed its distribution across various branches, emphasizing the diversity and variation within the UPRE2m populations (Fig. 2*D*). Within the high group, the conserved UPRE2m sequence is 5′-TACGTGTC-3′ (*SI Appendix*, Fig. S4).

**Fig. 3.**
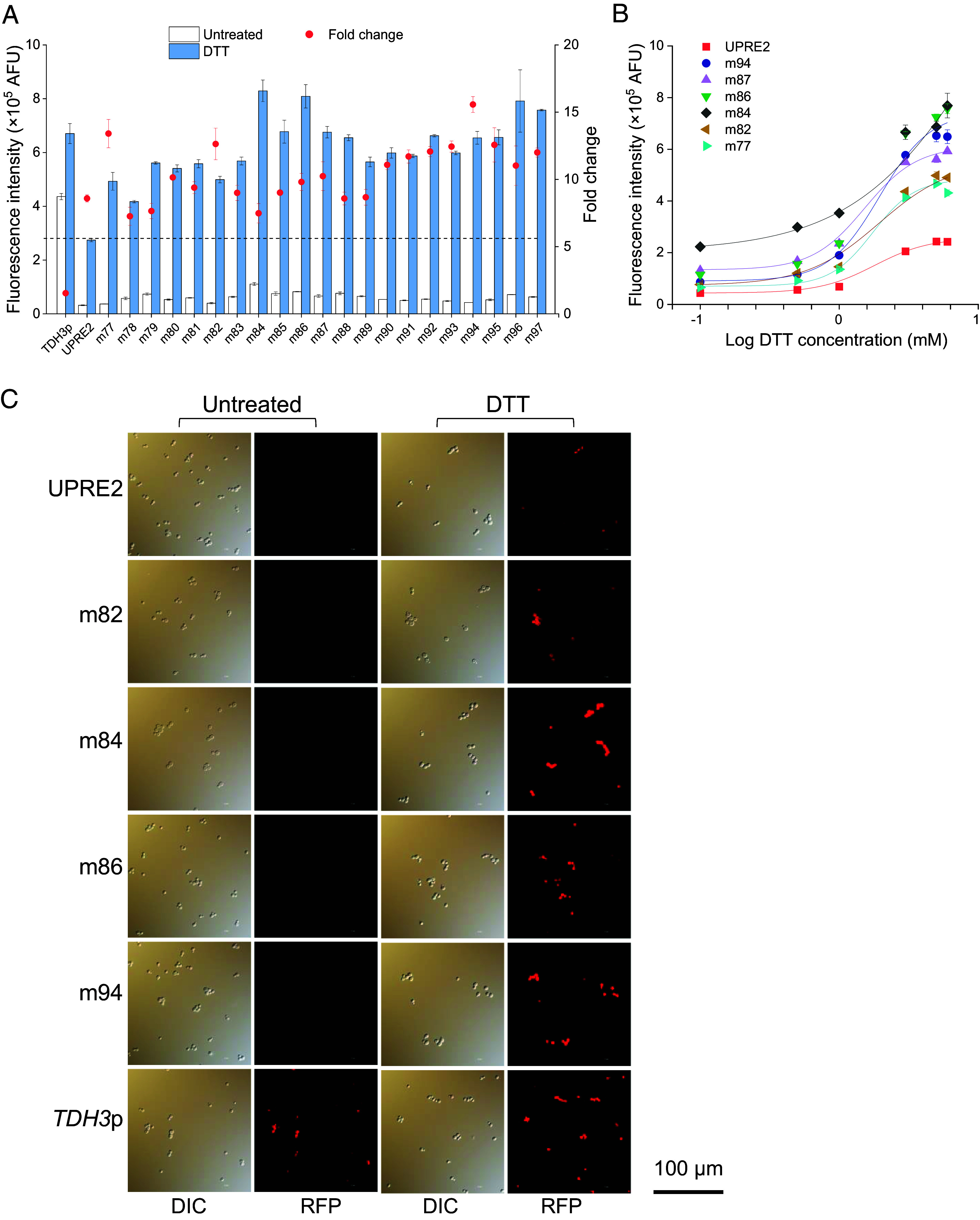
Response of the high-activity hybrid promoter UPRE2m-*TDH3*p CORE to DTT. (*A*) Response of hybrid promoter UPRE2m-*TDH3*p CORE to 5 mM DTT treatment. (*B*) Response of hybrid promoter UPRE2m-*TDH3*p CORE to varying DTT concentrations (0.1, 0.5, 1, 3, 5, and 6 mM). (*C*) Cell imaging of hybrid promoter UPRE2m-*TDH3*p CORE with high activity upon 5 mM DTT treatment. Data shown are mean values ± SDs of biological duplicates of single clones.

Tunicamycin (Tm) induces ER stress by inhibiting glycosylation (30). The response of UPRE2m-*TDH3*p CORE with high activity to Tm treatment was similar to its response upon DTT treatment, implying that UPRE2m effectively responds to ER stress triggered by different chemical reagents (Fig. 3*A* and *SI Appendix*, Fig. S5*A*). The efficacy of a gene regulatory system is determined by its genetic switch and its sensitivity; with sensitivity denoting the inducer concentration required to initiate gene expression (31). To assess the response sensitivity of UPRE2m, hybrid promoter UPRE2m-*TDH3*p CORE with high-activity elements (m84, m86, and m87) and wide-range response elements (m77, m82, and m94) were treated with different concentrations of DTT and Tm (Fig. 3*B* and *SI Appendix*, Fig. S5*B*). Of these, promoter m87-*TDH3*p CORE showed the highest sensitivity to DTT, whereas promoter m94-*TDH3*p CORE was most sensitive to Tm (*SI Appendix*, Table S2). Furthermore, the constitutive strong promoter *TDH3*p consistently exhibited high fluorescence levels in both untreated and DTT-treated conditions. Conversely, the hybrid promoter UPRE2m-*TDH3*p CORE (m82, m84, m86, and m94) exhibited a stronger response intensity in fluorescence imaging compared with UPRE2-*TDH3*p CORE under DTT treatment, further confirming the effectiveness of UPRE2m (Fig. 3*C*).

### Identification of UPRE2-Recognition Residues of Hac1.

**Fig. 4.**
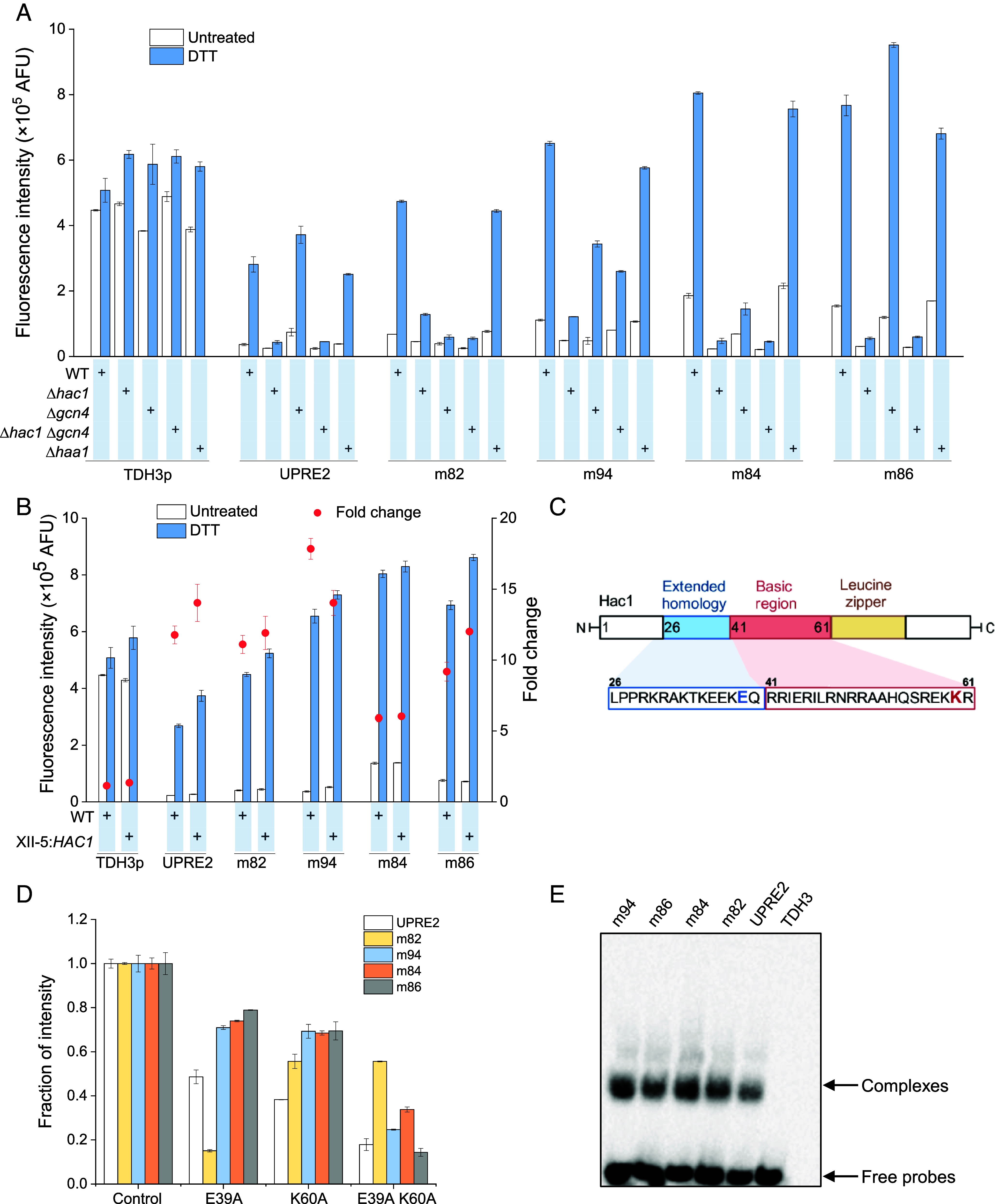
The amino acid residue K60 in the basic region of Hac1 is required for UPRE2m recognition. (*A*) Response of hybrid promoter UPRE2m-*TDH3*p CORE in transcription factor knockout strains. (*B*) Response of UPRE2m-*TDH3*p CORE in the *HAC1* overexpression strain. (*C*) Key amino acids in Hac1’s functional domains. (*D*) Relative responsiveness of hybrid promoter UPRE2m-*TDH3p* CORE in the Hac1-E39A, Hac1-K60A, and Hac1-E39A K60A strains, with the wild-type Hac1-complemented strain’s response activity set as a baseline at 1. (*E*) Binding of GST-Hac1 to UPRE2m as determined by EMSA. Data shown are mean values ± SDs of biological duplicates of single clones.

Hac1 binds to the UPRE upstream of the promoter, activating the transcription of UPR target genes (*SI Appendix*, Fig. S6). Previous studies have shown that UPRE2 is regulated by the transcription factor Hac1 and Gcn4. Gcn4 acts with or downstream of Hac1 (18). To determine how UPRE2m (m82, m94, m84, and m86) respond to Hac1 and Gcn4, the responsive expression cassette UPRE2m-*TDH3*p CORE-*mCherry* was integrated into *Δhac1*, *Δgcn4*, and *Δhac1Δgcn4* strains, using the *Δhaa1* strain as a control (Fig. 4*A*). Haa1 functions as a transcriptional activator in weak acid stress (32). The transcription factor deficient strains showed the following responses: the *Δhac1* strain had significantly reduced activity of UPRE2m-*TDH3*p CORE. Interestingly, the hybrid promoter with UPRE2 and m86 elements had a more potent response in the *Δgcn4* strain than that in the WT strain. It has been reported that an extension of the UPRE1 core sequence provides the necessary context for transcriptional function. The recognition of the extended context can be carried out by Hac1 without the cooperation of Gcn4 (18, 19). We speculated that the presence of extended flanking sequences may enhance the interaction between Hac1 and UPRE2, m86. In the *Δhaa1* strain, the response activity of UPRE2m-*TDH3*p CORE was similar to that in the WT strain. Besides, strains deficient in transcription factors exerted minimal influence on the activity of the *TDH3*p promoter. These findings suggested that Hac1 is the primary transcription factor regulating UPRE2m. Previous studies have demonstrated that alterations in the expression levels of key components in signal transduction pathways can influence both signal output and basal expression (33, 34). Therefore, we mutated Hac1 or fused the transactivation domain to its 3′ untranslated region to investigate its impact on signal transduction. Interestingly, the C-terminal 18-amino acid segment of Hac1 serves as an effective activation domain. In the Hac1-S238D strain, β-galactosidase expression was doubled (35). Gal4 or Med2 are frequently employed as transcription activation domains in yeast (36). The response of UPRE2-*TDH3*p CORE in the *Δhac1* strain, when complemented with Hac1-S238D or Hac1, was similar to that of the WT strain. However, when complemented with the Hac1-Gal4, the response significantly decreased (*SI Appendix*, Fig. S7). The 3′ sequence of *HAC1* pre-mRNA is crucial for Ire1-mediated splicing, leading to the formation of mature Hac1 mRNA (7). We speculated that the 3′ end of *HAC1* fused with the transcription activation domain sequence might affect the recognition or splicing of *HAC1* pre-mRNA by Ire1. Furthermore, we examined the impact of *HAC1* overexpression on the response elements. When overexpressing *HAC1*, hybrid promoters exhibited enhanced activity (Fig. 4*B*). Hence, *HAC1* overexpression enhances ER stress signal transduction and expands the signal output of the UPRE2m.

The basic leucine zipper transcription factor Hac1 consists of an extended homology domain, a DNA binding domain, a leucine zipper domain, and a C-terminal transactivation domain (Fig. 4*C*) (19). The phylogenetic alignment of Hac1 orthologs in fungi revealed a conserved region within both the extended homology and basic region, indicating a potential significance of these residues in DNA specificity (*SI Appendix*, Fig. S8). To identify the specific DNA residues recognized by Hac1, molecular docking was conducted between Hac1 and UPRE2m DNA. Residues Hac1-E39 in the extended homology region and Hac1-K60 in the basic DNA binding domain were identified as forming base-specific hydrogen bonds with UPRE2m. To verify the role of these residues in DNA recognition, Hac1 mutants were constructed, in which the recognition residues were mutated to alanine. The response activity of the hybrid promoter was reduced in both Hac1 mutant strains but was more pronounced in the Hac1-K60A. Additionally, the response activity of the hybrid promoter was further reduced in the Hac1-E39A K60A mutant strain (*SI Appendix*, Fig. S9). This indicates that Hac1-K60 plays a critical role in the recognition and binding of UPRE2.

Compared with the control, the relative activity of the hybrid promoter differed in the Hac1 mutant strains (Fig. 4*D*). Furthermore, the specificity of the base-specific hydrogen bonds formed between Hac1-K60 and different UPRE2m molecules were located within the conserved sequence of UPRE2m (*SI Appendix*, Fig. S10 *A*–*E*). Specifically, the base-specific residues of UPRE2, m94, m82, and m86 reside within the conserved sequence 5′-ACGTGTC-3′. The base-specific residue of m84 is found in the reverse complementary sequence, 5′-GACACGT-3′. Remarkably, Hac1-K60 forms hydrogen bonds with two bases within the conserved sequence 5′-ACGTGTC-3′ of m86. However, it remains to be determined whether this interaction is associated with the higher basal expression activity of m86. Moreover, saturation mutagenesis was applied to Hac1-K60, revealing that none of the mutations increased the response activity of UPRE2m-*TDH3*p CORE (*SI Appendix*, Fig. S11). The activity of the hybrid promoters in the Hac1-K60A strain ranged between 38% and 69% of that in the control strain. Notably, the hybrid promoters m82-*TDH3*p CORE and m84-*TDH3*p CORE maintained relative high activity in the Hac1-K60R mutant strain. Mutation of the K60 residue to an aromatic amino acid almost completely abolished the activity of the hybrid promoter. Similarly, the Hac1-K60F mutant failed to the survival of the *Δhac1* strain in media containing Tm and DTT (*SI Appendix*, Fig. S12). To analyze the DNA binding properties of Hac1, we conducted an electrophoretic mobility-shift assay (EMSA) using purified glutathione S-transferase-Hac1(GST-Hac1) (*SI Appendix*, Fig. S13) and biotin-labeled oligonucleotides (*SI Appendix*, Table S6). Mobility retardation was observed with biotinylated UPRE2 and UPRE2m probes, but not with the control TDH3 probe, indicating Hac1’s specific binding to these probes (Fig. 4*E* and *SI Appendix*, Fig. S14*A*). The GST does not interfere with the specific binding of GST-Hac1 to UPRE2m (*SI Appendix*, Fig. S14*B*). Hac1 exhibited higher affinity binding to m82, m84, m86, and m94, compared with the native UPRE2. Considering the variations in nucleotide composition among different UPRE2m sequences, the higher binding affinity between Hac1 and UPRE2m may contribute to the enhancement of both basal expression and response activity of the hybrid promoter UPRE2m-*TDH3*p CORE.

### Modular Assembly Optimized UPRE2m Performance.

**Fig. 5.**
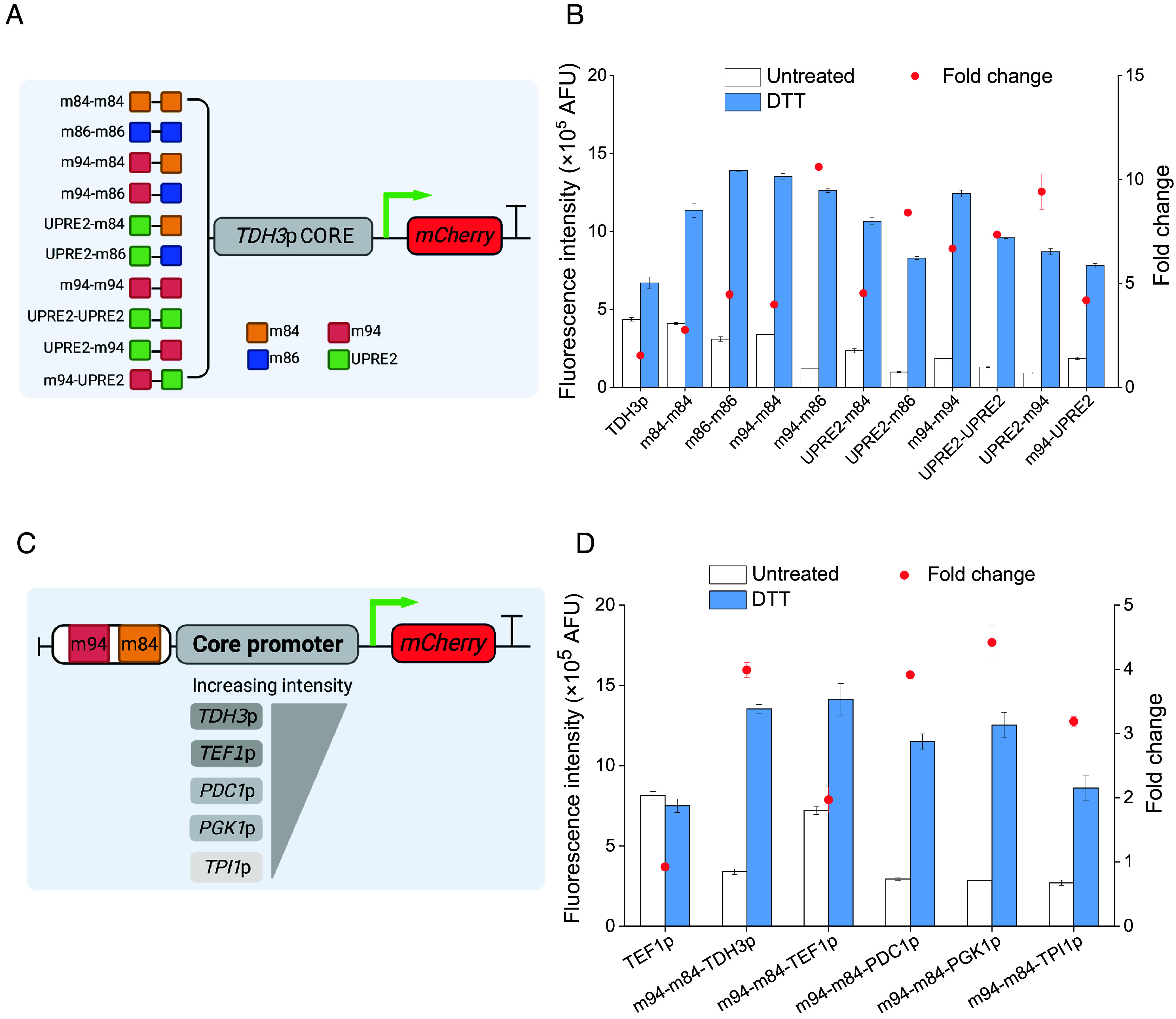
Modular assembly of UPRE2m. (*A* and *B*) Response of the hybrid promoter 2×UPRE2m-*TDH3*p CORE to 5 mM DTT treatment. (*C* and *D*) The element m94-m84 was assembled with core regions of different promoters and their responses to 5 mM DTT treatment. Data shown are mean values ± SDs of biological duplicates of single clones.

Regulatory regions with multiple upstream activating sequences binding the same transcription factor have been shown to amplify the transcriptional capacity of promoters (37). To enhance the response activity of the elements further, different elements were concatenated and inserted upstream of the *TDH3*p CORE (Fig. 5*A*). Various combinations were made between elements with strong response activity (m84 and m86), elements with a broad response dynamic range m94 and UPRE2. However, arranging strong response active elements in tandem increased the basal expression level of the hybrid promoter, but reduced its range of response. A similar observation was made in a previous study, where host cells with high-copy plasmids exhibited a reduced fluorescence induction range compared to strains having integrated plasmids (38). Among all tandem arrangements tested, m94-m86 and m94-m84 resulted in the broadest range of response and the highest response activity for the promoter, respectively (Fig. 5*B*). The UPRE2-m94 combination in the promoter exhibited a wider dynamic response as well. It has been reported that both the binding affinity and the flanking sequence around the TATA box influence promoter activity (39). To explore whether 2×UPRE2m could serve as an assembly module, m94-m84 was subsequently inserted upstream of the core region of different strong promoters (*PDC1*p, *PGK1*p, *TEF1*p, and *TPI1*p) (Fig. 5*C*). The response activity of the hybrid promoter surpassed that of the constitutive promoter *TEF1*p under DTT treatment (Fig. 5*D*). These results indicated that UPRE2m can serve as a versatile module for assembly with different promoters.

To investigate the adaptability of these elements in other strains, the response of UPRE2m in the BY4742 strain was evaluated. Active UPRE2m remained its responsiveness to DTT-induced ER stress in the BY4742 strain (*SI Appendix*, Fig. S15). Additionally, active UPRE2m was sensitive to DTT-induced ER stress (*SI Appendix*, Fig. S16).

### Exploitation of the UPRE2m as a Promoter-Responsive Enhancer Element.

**Fig. 6.**
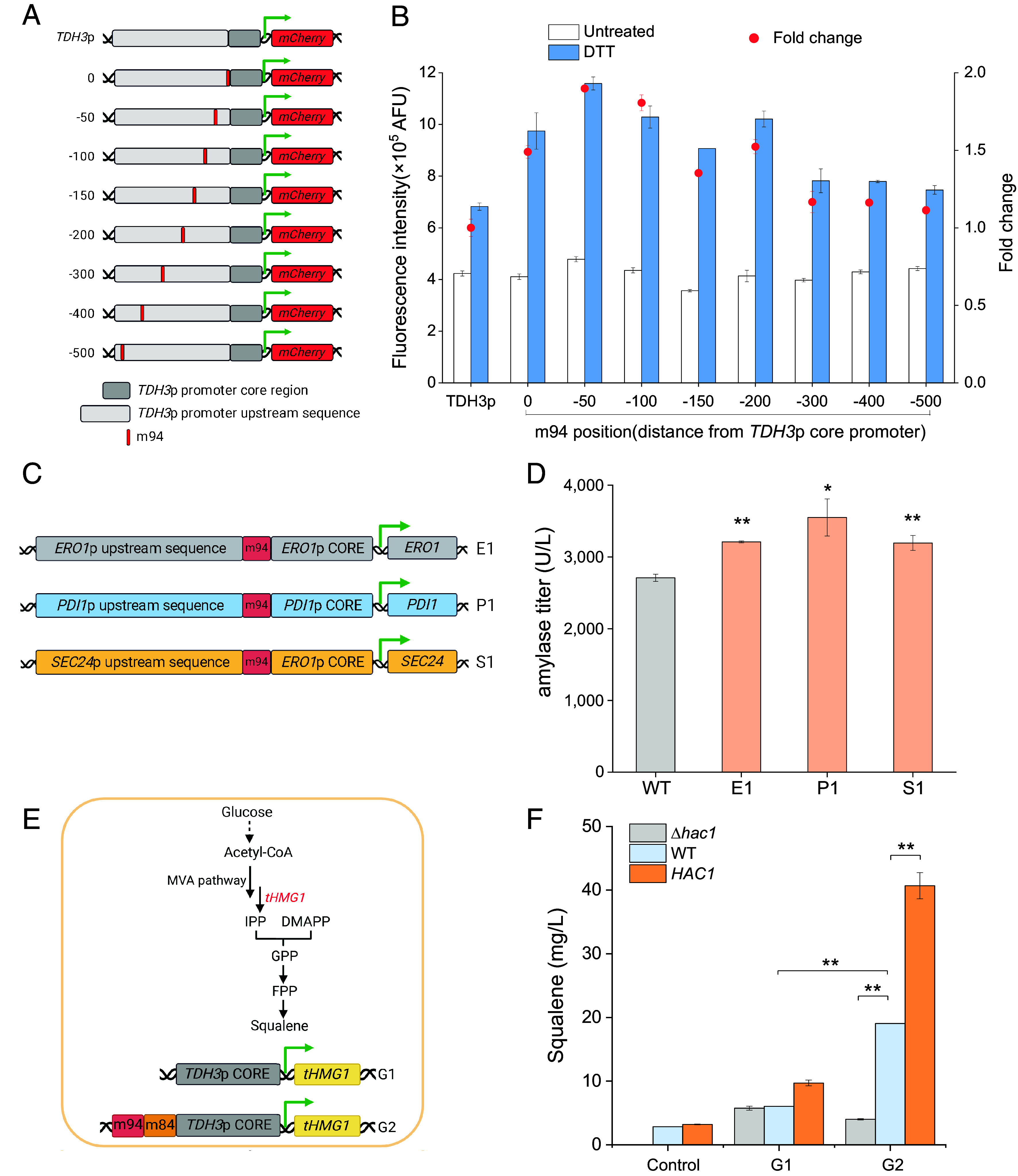
Position effects of UPRE2m and UPRE2m application in the synthetic responsive promoter in *S. cerevisiae.* (*A* and *B*) Influence of m94 positioning on *TDH3*p activity. (*C* and *D*) Effect of m94 insertion upstream of the target promoter on α-amylase production. (*E* and *F*) Impact of promoters (G1: *TDH3*p CORE, G2: m94-m84-*TDH3*p CORE) on regulating *tHMG1* expression and subsequent squalene production in *Δhac1*, WT, and *HAC1* overexpression strains; strains without *tHMG1* expression cassettes served as controls; strains were cultured in YPD medium. Data shown are mean values ± SDs of biological duplicates or triplicates of single clones. The statistical significance was determined by a two-tailed homoscedastic (equal variance) *t* test. *P < 0.05; **P < 0.01.

UPRE2m confers responsiveness to promoters. We further investigated how the position of this element within the promoter influenced gene expression. The element m94 with the broadest response dynamic range was inserted at different positions upstream of the *TDH3*p CORE ranging from −500 bp to 0 bp. Upon DTT treatment, constructs containing m94 exhibited enhanced promoter activity compared with the control. As the distance between the insertion site and the core promoter increased, there was a general decline in mCherry expression (Fig. 6 *A* and *B*). A similar phenomenon was observed in previous study. The correlation between expression levels and site positions follows a unique jagged function involving TF binding sites and their contextual combinations (40).

The interaction between UPRE2m and Hac1 holds the potential to function as a responsive enhancer for promoters. These UPRE2m were further validated in practical applications. The m94 was used as a regulatory element for specific target genes in the recombinant α-amylase expression strains. Selected targets were those reported to enhance α-amylase production or pivotal for protein secretion. These included *ERO1* and *PDI1*, involved in disulfide bond formation of proteins, and *SEC24*, essential for cargo selection during vesicle transition from the ER to the Golgi apparatus (41–43). The 150 to 250 bp region upstream of the target coding sequence was designated as the core promoter region. The m94 element was then inserted directly upstream (0 bp) of this core region of the target promoter (Fig. 6*C*). Strains E1, P1, and S1, added with m94 in their target promoters, showed a significant increase in amylase production compared with the WT (Fig. 6*D*). This indicates that m94, acting as a responsive enhancer element, plays a role in regulating targets and increase recombinant protein production.

To evaluate the potential of the response elements in metabolic pathway modulation, they were used to regulate truncated HMG-CoA reductase (*tHMG1*), aiming to enhance squalene synthesis. Squalene is a key precursor in the biosynthesis of various triterpenoids and steroids, with applications across the pharmaceutical, nutraceutical, and cosmetic industries (44, 45). *S. cerevisiae* naturally synthesizes squalene via the mevalonate (MVA) pathway (Fig. 6*E*). Overexpressing *tHMG1* can alleviate feedback inhibition of mevalonate, thereby optimizing the MVA pathway (46). The level of *tHMG1* expression is directly linked to squalene accumulation (47). Expanding the ER increases its protein synthesis capacity and provides more space to accommodate proteins, resulting in a significant increase in terpene production (48). To evaluate the impact of the response elements on metabolic pathway modulation, we used different promoters (G1: *TDH3*p CORE, G2: m94-m84-*TDH3*p CORE, G3: *TDH3*p, G4: m94 positioned at the −50 site upstream of *TDH3*p CORE within *TDH3*p) to control *tHMG1* expression in the *Δhac1*, WT, and *HAC1* overexpression strains. The expression cassettes for *tHMG1* were chromosomally integrated into these strains. Compared with using G1, G2 resulted in higher squalene production. Furthermore, G2 showed significantly higher squalene production in the WT strain than in the *Δhac1* strain, with an even greater increase in the *HAC1* overexpression strain (Fig. 6*F* and *SI Appendix*, Fig. S17). This suggested that the m94-m84-*TDH3*p CORE promoter was induced under stress conditions, leading to increased *tHMG1* expression and subsequently enhanced squalene production, especially when Hac1 levels were elevated. The *tHMG1* was also expressed using the constitutive strong promoter *TDH3*p (G3) and the hybrid *TDH3*p promoter with an m94 insertion (G4) (*SI Appendix*, Fig. S18). Strains harboring G3 or G4 expression cassettes exhibited high squalene production, attributed to the elevated expression levels driven by *TDH3*p and its hybrid variant, both of which have high basal expression capabilities. Adding DTT to the medium further increased squalene production (*SI Appendix*, Fig. S18), likely because of the enhanced strength of the *TDH3*p under DTT treatment (Fig. 6*B*). The hybrid promoter outperformed the TDH3p promoter under DTT treatment, indicating its responsiveness to ER stress, although the difference was modest, possibly due to the already high basal expression level. These findings emphasize the significance of optimal expression intensity of key enzymes in metabolic pathways. Furthermore, it highlights the efficiency of an ER stress-responsive hybrid promoter in coordinating the MVA biosynthetic pathway, showcasing its potential for metabolic engineering applications.

## Conclusions

In summary, our work focused on developing additional UPRE2 variants and analyzing their interactions with related transcription factors. Through multiple rounds of screening and validation, we identified m84 as the most active element, displaying a response activity 3.72 times higher than native UPRE2. Additionally, element m94, possessing the widest dynamic range, exhibited a 103% increase in response range compared with the native UPRE2. Grouping these elements by response activity revealed a potential correlation between high activity UPRE2m and DNA sequence free energy. Notably, the response of UPRE2 was influenced by its nucleotide composition and length of its flanking sequences. Evaluations in transcription factor deficient strains highlighted Hac1 and Gcn4 as pivotal in UPRE2m regulation. Overexpressing *HAC1* enhanced the response activity of UPRE2m. Furthermore, we found that the basic DNA binding domain K60 of Hac1 was required for UPRE2m recognition. Hac1-K60 formed specific hydrogen bonds with core sequences across different UPRE2m. This finding provides insights into the mechanism by which Hac1 binds to UPRE2 DNA target sites, deepening our understanding of the foundational process. The UPRE2m can act as a responsive module within the promoter, enhancing the stress-induced expression of target genes and thereby increasing product production. Based on the interaction between UPRE2m and Hac1, additional orthogonal elements can be developed as genetic tools for robust target regulation.

## Materials and Methods

### Plasmid and Strain Construction.

The plasmids, strains, and primers used in this study were listed in *SI Appendix*, Tables S3–S5, respectively. The pUC57-UPRE2-*TDH3*p-EforRed-*CYC1*t plasmid was synthesized by GenScript. The EforRed and GFP were connected by a (GGGGS)×3 linker. The promoter fragments were amplified from the yeast genome. Tandem UPRE2 elements were obtained by PCR with primers containing corresponding sequences. The fragments were ligated to the plasmid backbone using the Gibson cloning method (49), and the products were introduced into *Escherichia coli* DH5α.

Gene knockout or integration was carried out in the IMX581 (50) and MSBP003 strains (51) using the CRISPR/cas9 system. Yeast cells were transformed with the auxiliary plasmid and DNA repair fragments and spread on SD-URA plates to allow colony formation. Transformants were streaked on 5-fluoroorotic acid (5′-FOA) plates to remove the auxiliary plasmid. Yeast transformation was accomplished by using the lithium acetate/single-stranded carrier DNA/PEG method (52).

### Media and Cell Cultures.

All *E. coli* strains were cultured in LB medium containing 10 g/L tryptone, 5 g/L yeast extract, and 10 g/L NaCl. Then, 100 mg/L ampicillin was added to the medium for selection purposes if necessary. The genotypes of *S. cerevisiae* strains are described in *SI Appendix*, Table S4. The medium used for cultivating yeast strains was tailored according to the auxotrophic requirements of the yeast cells. *S. cerevisiae* strains were grown at 30 °C and 200 rpm. *E. coli* strains were grown at 37 °C and 250 rpm. Two percent of agar powder was added to the corresponding liquid medium when preparing agar plates.

YPD medium contains 20 g/L peptone, 10 g/L yeast extract, and 20 g/L glucose. YPE medium contains 20 g/L peptone, 10 g/L yeast extract, 0.5 g/L glucose, and 10 g/L ethanol. SD-URA medium contains 0.77 g/L complete supplement mixture (CSM, without Uracil), 1.7 g/L yeast nitrogen base (without amino acids and (NH_4_)_2_SO_4_), 5.0 g/L (NH_4_)_2_SO_4_, 20 g/L glucose (pH adjusted to 5.5 to 6.0). SD medium supplemented with 0.8 g/L 5′-FOA was used to remove the *URA3*-based plasmid. Yeast strains were cultivated in SD-2×SCAA medium for α-amylase production. SD-2×SCAA medium contains 20 g/L glucose, 1.70 g/L yeast nitrogen base without amino acids and ammonium sulfate, 5.0 g/L (NH_4_)_2_SO_4_, 190 mg/L arginine, 400 mg/L aspartic acid, 1,260 mg/L glutamate, 130 mg/L glycine, 140 mg/L histidine, 290 mg/L isoleucine, 400 mg/L leucine, 440 mg/L lysine, 108 mg/L methionine, 200 mg/L phenylalanine, 220 mg/L threonine, 40 mg/L tryptophan, 52 mg/L tyrosine, 380 mg/L valine, 1 g/L bovine serum albumin (BSA), 5.4 g/L Na_2_HPO_4_, and 8.56 g/L NaH_2_PO_4_·H_2_O (pH 6.0). A total of 40 mg/L Uracil was added to SD-2×SCAA medium when necessary.

### Fluorescence Intensity Measurement.

For chemical stress induction, colonies from fresh plates were incubated in SD-URA medium and allowed to grow overnight at 30 °C and 200 rpm. The seed cultures were subsequently inoculated into a fresh SD-URA medium with an initial cell density of OD_600_ of 0.1 [OD_600_ of 0.1 corresponds to approximately 1 × 10^6^ cells/mL (52)]. When the cells grown to an OD_600_ between 0.3 and 0.4, chemical reagents were added and the cells were treated for 4 h.

For fluorescence intensity measurement, the untreated and stress-induced cells were collected by centrifugation and subsequently washed twice with phosphate-buffered saline (PBS). The cell pellets were resuspended with PBS. Two hundred microliters of the cell suspension was loaded into a 96-well black transparent plate. Light with wavelengths of 532 nm and 485 nm, respectively, was used to excite mCherry and GFP. The emission wavelengths detected for mCherry and GFP were 610 nm and 520 nm, respectively. The fluorescence intensity of mCherry and GFP was quantified using a microplate reader (SpectraMax iD3, Molecular Devices). Cells without expression of fluorescent protein were used as negative control. The fluorescence values were normalized to the OD_600_.

For cell imaging, both untreated strains and those induced with 5 mM DTT were washed twice with PBS. The fluorescence emitted by mCherry was then captured using a fluorescence microscope (H600L, Nikon).

### Cell Viability Assay.

The overnight strain culture was diluted in a 10-fold gradient, using a concentration of OD_600_ = 0.1 as the starting point. Then, 3 μL of the diluted sample was spotted onto plates containing either DTT or Tm.

### Calculation of DNA Stability.

The stability of double-stranded DNA was determined by the sum of the free energy of its constituent base-paired dinucleotides which was primarily influenced by factors such as the GC content and the types and orientations of the base pairs in the DNA molecule (53). The energy values for the 16 dinucleotide steps were derived from the unified parameters obtained from melting studies on 108 oligonucleotides (54).

### Sequence Alignments and Phylogenetic Trees.

The sequence alignment was generated with Clustal Omega and visualized through Jalview. Phylogenetic trees were constructed using both Jalview and the website https://itol.embl.de/.

### α-Amylase Quantification.

Strains were cultured in SD-2×SCAA medium at 30 °C and 200 rpm for 96 h. For protein quantification, 500 µL of cell cultures was centrifuged at 12,000 × g for 1 min to separate the supernatant from the cell pellet. The supernatant was used to measure extracellular α-amylase production. The activity of α-amylase was measured using an α-amylase assay kit (K-CERA, Megazyme). A commercial *Aspergillus oryzae* α-amylase (Sigma-Aldrich) was used as a standard.

### Expression and Purification of GST-Hac1 Variants.

GST-Hac1 was expressed in *E. coli* BL21(DE3) by incubation with 0.3 mM isopropyl β-D-thiogalactoside (IPTG) for 15 h at 20 °C in LB medium containing 100 μg/mL ampicillin. The cells were harvested and lysed by sonication in cold PBS buffer with a protease inhibitor cocktail (Biosharp). The GST-tagged protein GST-Hac1 was purified using a GSTrap FF column (Cytiva), following the manufacturer’s protocol. Protein concentrations were determined by measuring absorbance at 280 nm and applying the respective molar extinction coefficients.

### Electrophoretic Mobility-Shift Assay.

All probes used are listed in *SI Appendix*, Table S6. The 5′ end biotinylated complementary oligonucleotide pairs (Sangon) were annealed to create double-stranded, biotin-labeled probes. This process involved mixing in a buffer (10 mM Tris–HCl, pH 8.0, and 1 mM EDTA), at 99 °C for 5 min, and then allowing them to cool slowly to room temperature. Unlabeled complementary oligonucleotide pairs were similarly annealed to produce double-stranded competitor probes. All EMSA experiments were performed using a Chemiluminescent EMSA Kit (Beyotime), following the manufacturer’s protocol. A purified GST recombinant protein (D610271, Sangon) was used as a control, obtained from the supplier.

### Molecular Docking.

The Hac1 structure was modeled using AlphaFold2, and the double helical secondary structure of UPRE2m DNA was generated via the website https://scfbio-iitd.res.in/software/drugdesign/bdna.jsp#. Molecular docking predictions of Hac1 with UPRE2m DNA were performed using HADDOCK 2.4, available at https://wenmr.science.uu.nl/haddock2.4/. The docking results between Hac1-K60 and UPRE2 as well as UPRE2m were analyzed and visualized using PyMOL.

### Extraction and Quantification of Squalene.

To extract squalene, 500 μL of cultured cells and 1 mL of ethyl acetate were combined with 0.7 g of zirconia beads (0.5 mm in diameter) in 2 mL microcentrifuge tubes and ran on a cell homogenizer (Allsheng, Bioprep-24R) at 6.5 m/s for 2 min (55). The mixed solution was centrifuged at 13,000 rpm for 10 min, and the upper organic phase was filtered for HPLC analysis. Squalene was analyzed by an HPLC system (Shimadzu) with loading of extraction to an Aminex HPX-87H column (Bio-Rad). Acetonitrile was used as the mobile phase to flow in the HPLC system at a rate of 1.2 mL/min at 25 °C (56).

## Supplementary Material

Appendix 01 (PDF)

## Data Availability

All study data are included in the article and/or *SI Appendix*.
