# Supplementary material for: Tailored UPRE2 variants for dynamic gene regulation in yeast
Source: Proc Natl Acad Sci U S A. 2024 Apr 30;121(19):e2315729121. doi: 10.1073/pnas.2315729121 (PMC11087760; doi:10.1073/pnas.2315729121)
Supplement: Supplementary file 1 — Appendix 01 (PDF) [file pnas.2315729121.sapp.pdf]

Supporting Information for

Tailored UPRE2 Variants for Dynamic Gene Regulation in Yeast

Chufan Xiao[a,1], Xiufang Liu[a,1], Yuyang Pan[a], Yanling Li[a], Ling Qin[a], Zhibo Yan[a], Yunzi Feng[a], Mouming Zhao[a] and Mingtao Huang[a,2]

[a]School of Food Science and Engineering, South China University of Technology, Guangzhou, 510641, China
[1]C.X. and X.L. contributed equally to this work.
[2] To whom correspondence should be addressed. Email: huangmt@scut.edu.cn.

This file includes:

Fig. S1 to S18
Table S1 to S6

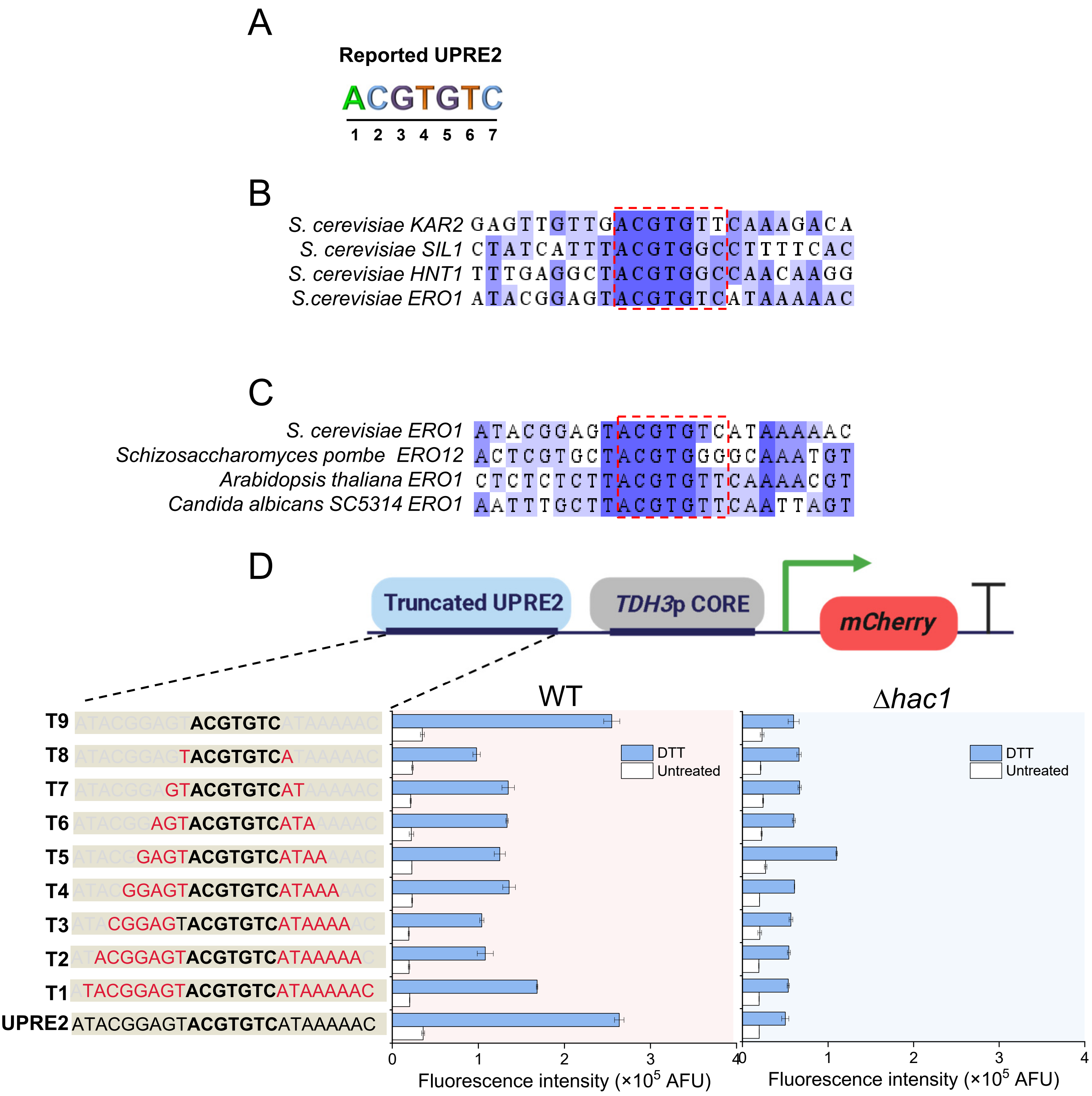

Fig. S1. UPRE2 sequence conservation. (A) Reported core sequence of UPRE2. (B) Alignment of sequences containing UPRE2 in known Hac1p target promoters. (C) Alignment of homologous promoter sequences of *ERO1*p. The reported core sequence of UPRE2 (5'-ACGTGTC-3') is indicated by a red dashed box. Conserved 5′ and 3′ flanking nucleotides are indicated by blue shading. (D) Response of hybrid promoter *TDH3*p CORE with the truncated UPRE2 to 5 mM DTT treatment in WT and Δ*hac1* strains. Data shown are mean values ±SDs of biological duplicates of single clones.

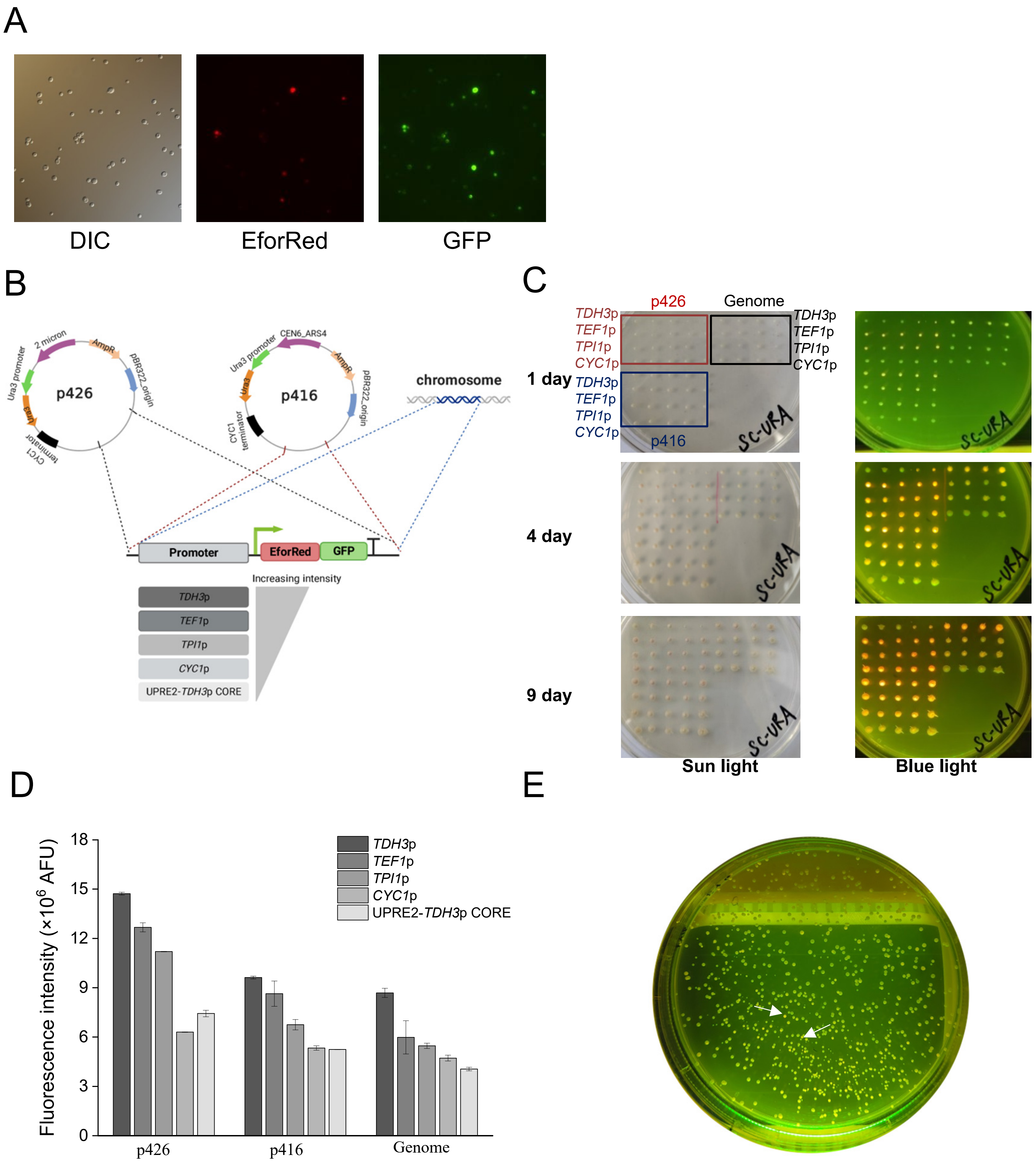

Fig. S2. Expression analysis of reporter protein EforRed-GFP. (A) Fluorescence microscopy imaging of EforRed-GFP. (B-C) Expressing reporter protein using promoters with different expression strengths (*TDH3*p>*TEF1*p>*TPI1*p>*CYC1*p) on different vectors: high-copy plasmid p426, low-copy plasmid p416, and the genome. (D) The fluorescence intensity of GFP. (E) Visualization of the mutants under blue light. Clones with higher fluorescence intensity are indicated by white arrows. Data shown are mean values ±SDs of biological duplicates of single clones.

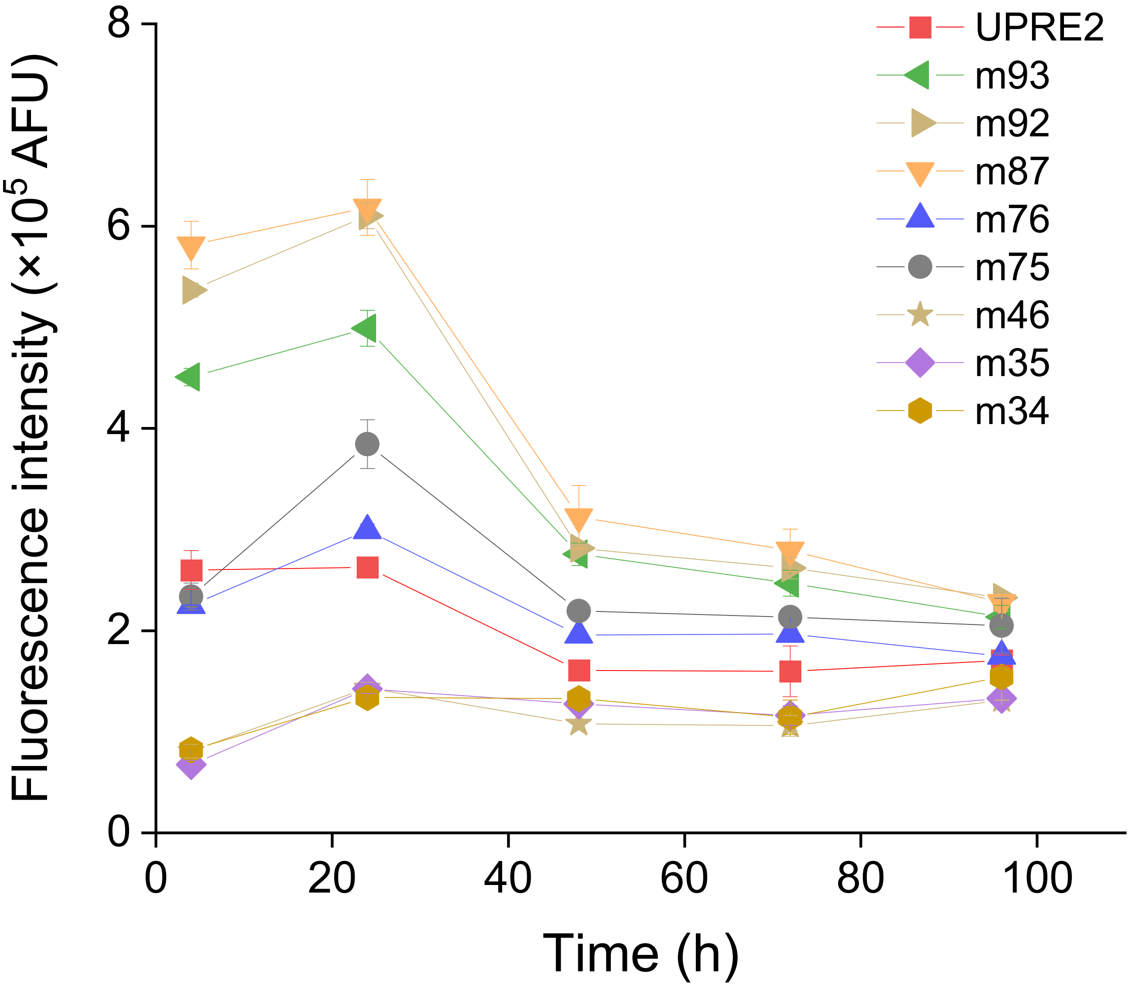

Fig. S3. Time-course response of hybrid promoter UPRE2m-*TDH3*p CORE to 5mM DTT treatment. Data shown are mean values ±SDs of biological duplicates of single clones.

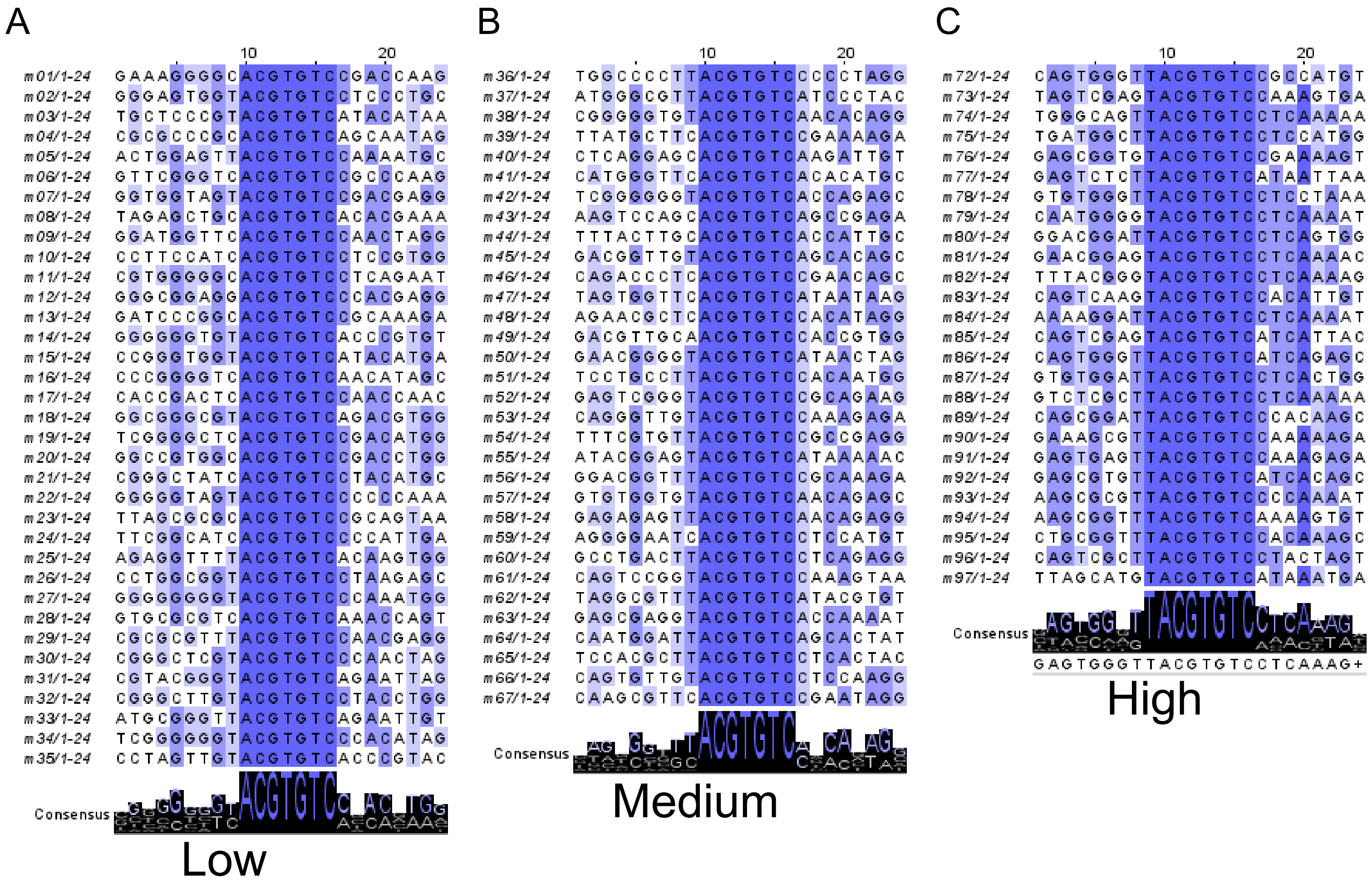

Fig. S4. Sequence alignment of different UPRE2m groups exhibiting varied activities.

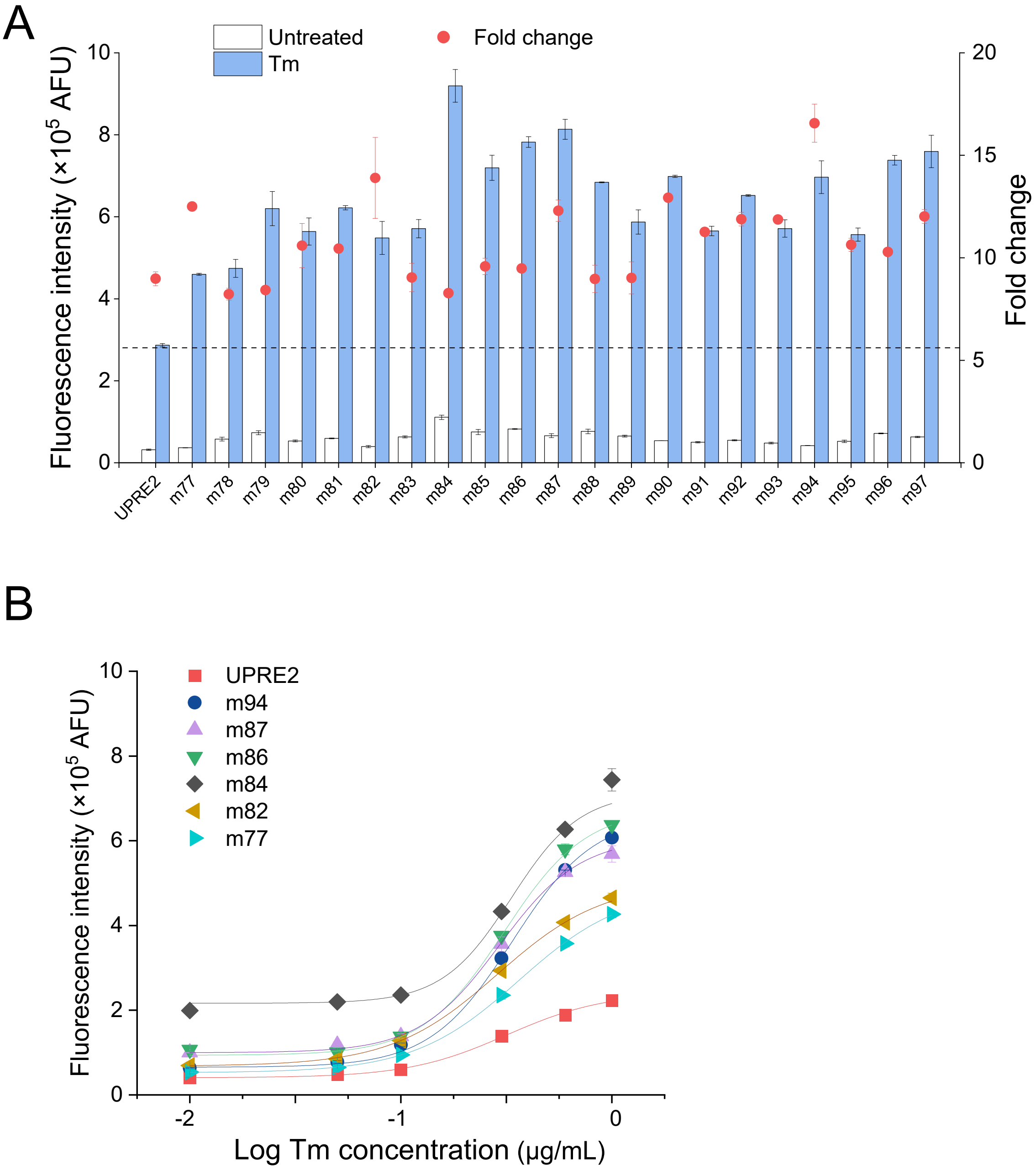

Fig. S5. Response of high activity hybrid promoter UPRE2m-*TDH3*p CORE to tunicamycin (Tm). (A) Response of hybrid promoter UPRE2m-*TDH3*p CORE to 0.6 μg/mL Tm treatment. (B) Response of hybrid promoter UPRE2m-*TDH3*p CORE to different concentrations of Tm treatment (0.01, 0.05, 0.1, 0.3, 0.6, 1 μg/mL). Data shown are mean values ±SDs of biological duplicates of single clones.

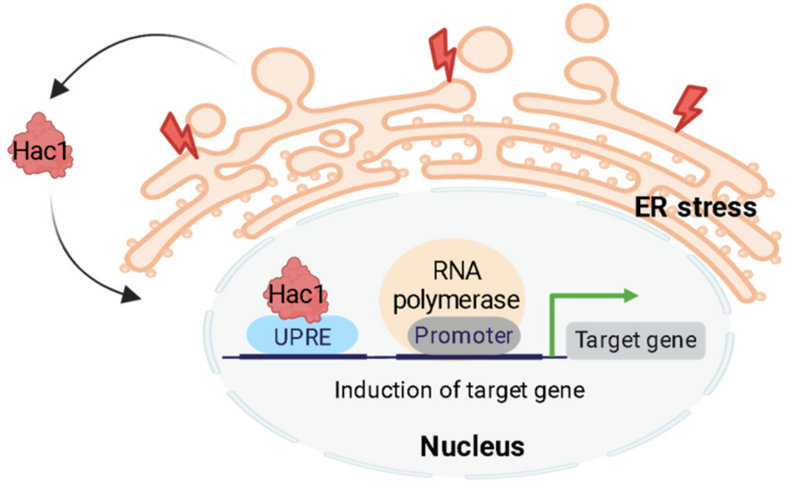

Fig. S6. Interaction schematic between Hac1 and UPRE in *S. cerevisiae.*

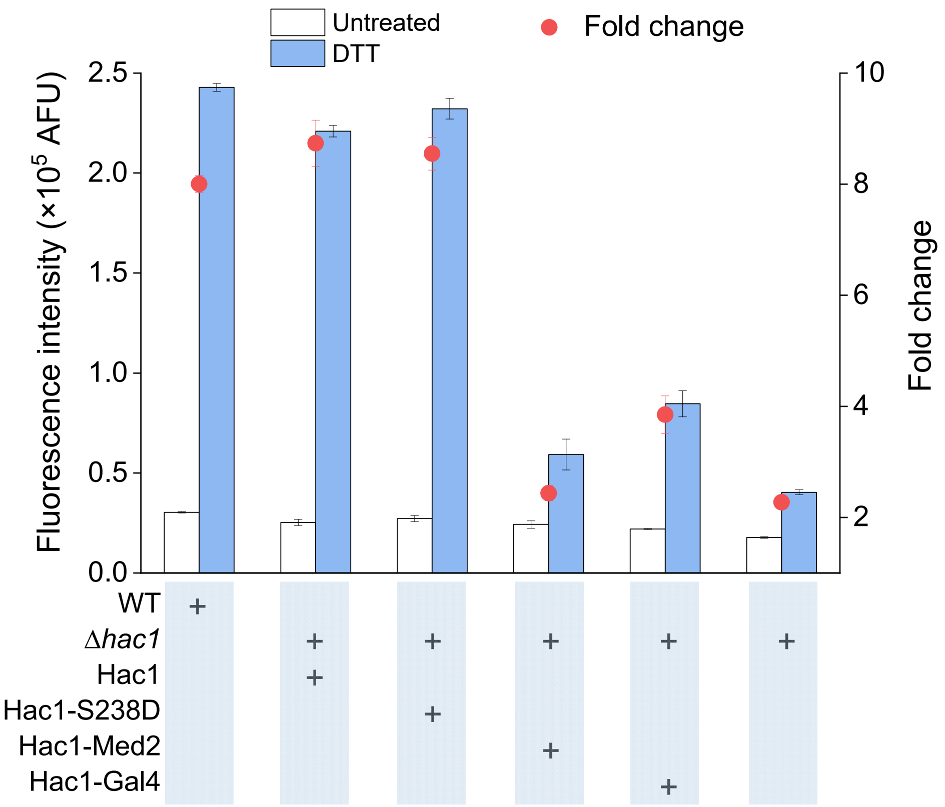

Fig. S7. Response of the hybrid promoter UPRE2-*TDH3*p CORE to 5 mM DTT treatment in Hac1-S238D, Hac1-Med2 and Hac1-Gal4 strains. Data shown are mean values ±SDs of biological duplicates of single clones.

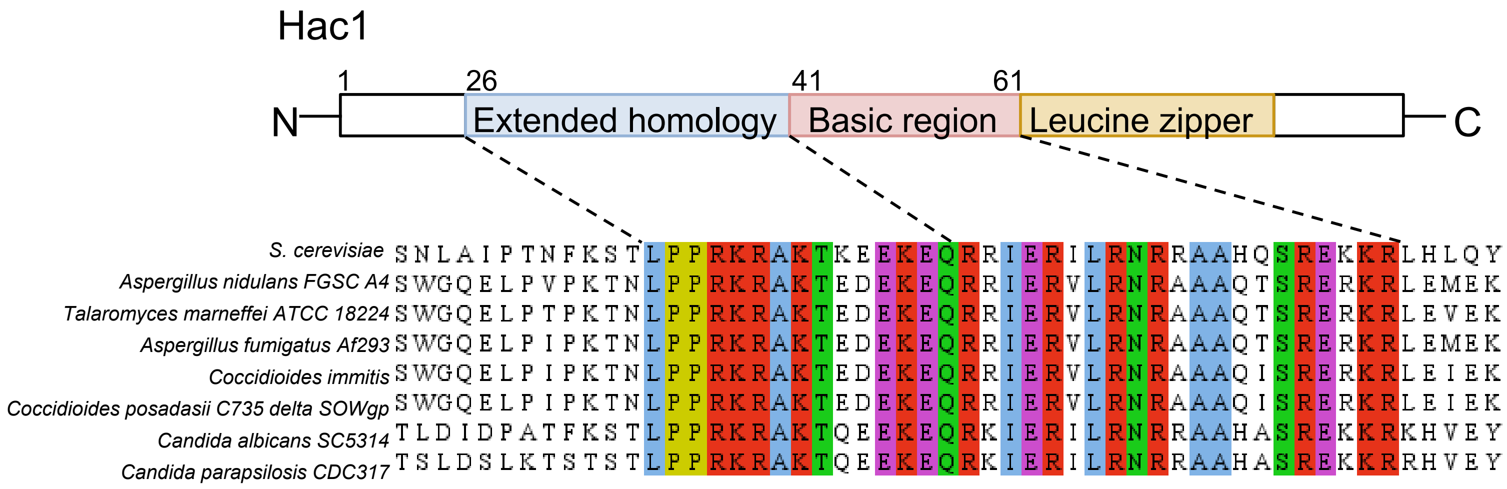

Fig. S8. The phylogenetic alignment of homologous Hac1 across fungal species. Amino acid residues with 100% conservation are indicated by shading.

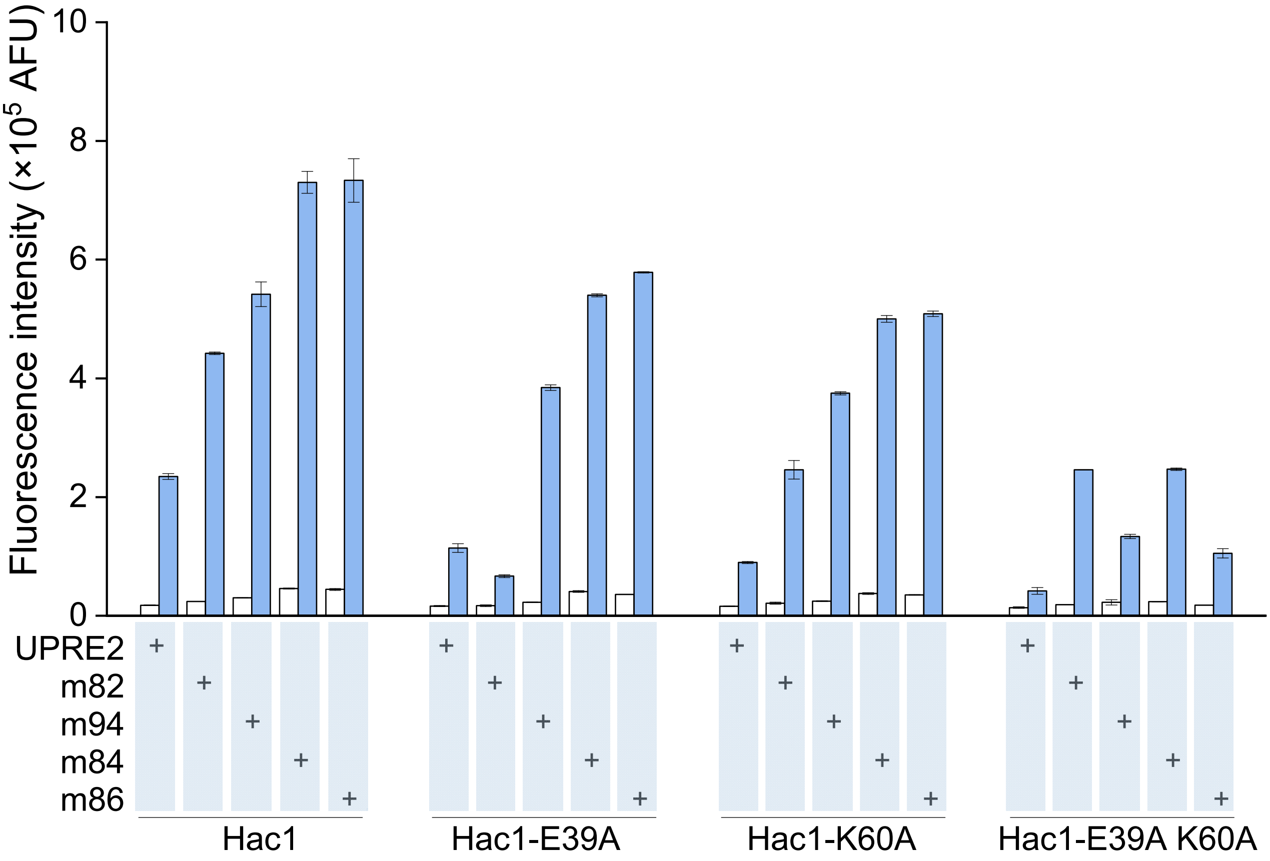

Fig. S9. Response of hybrid promoter UPRE2m-*TDH3*p CORE to 5 mM DTT treatment in Hac1-E39A, Hac1-K60A , Hac1-E39A K60A strains. Data shown are mean values ±SDs of biological duplicates of single clones.

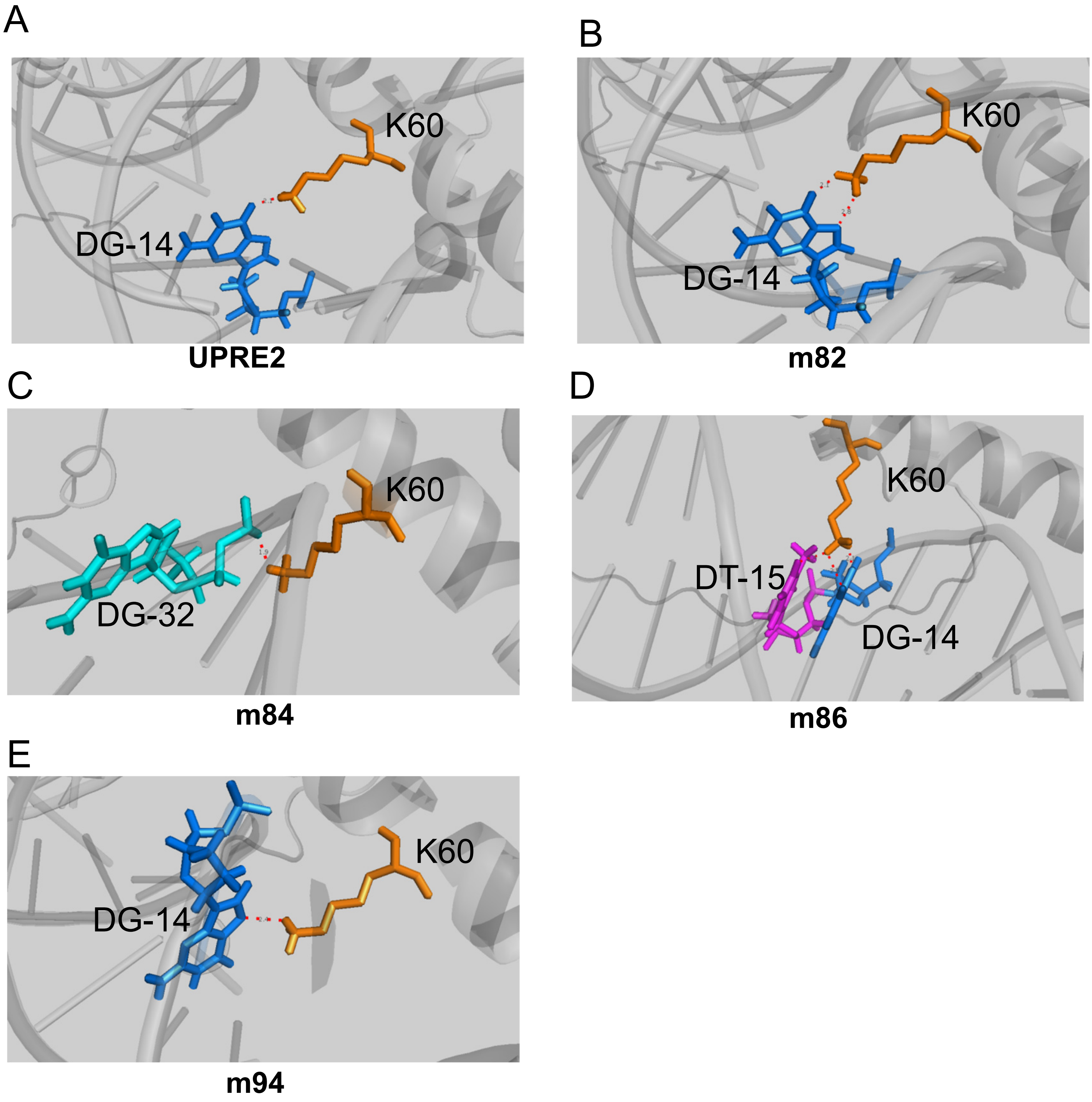

Fig. S10. Molecular docking of Hac1-K60 to UPRE2m. (A-E) Specific hydrogen bonding between Hac1-K60 and UPRE2m. Hydrogen bonds are indicated by red dashed lines.

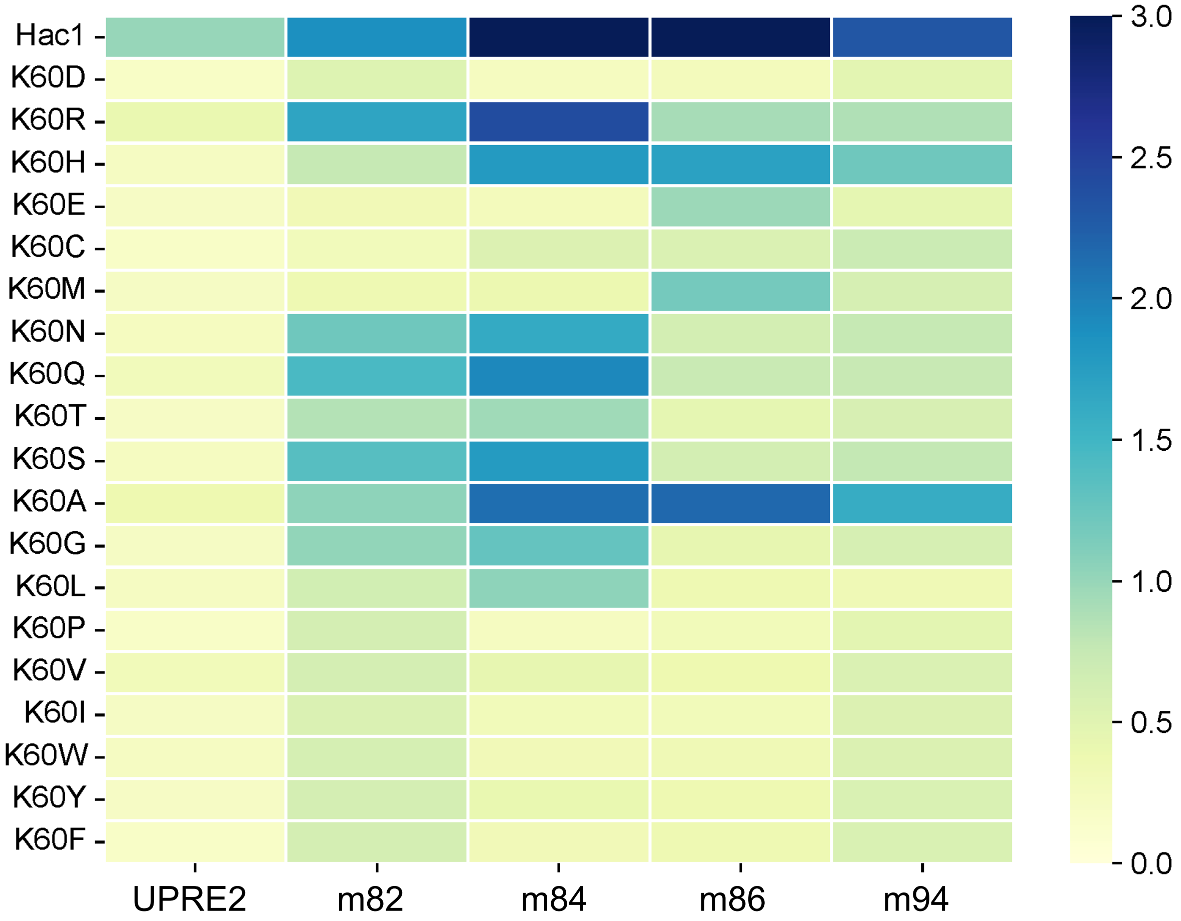

Fig. S11. Relative responsiveness of hybrid promoter UPRE2m-*TDH3*p CORE to 5 mM DTT in Hac1-K60 mutant strains, with the response activity of the UPRE2-*TDH3*p CORE in the control strain (Hac1) set at 1. Data shown are mean values of biological duplicates of single clones.

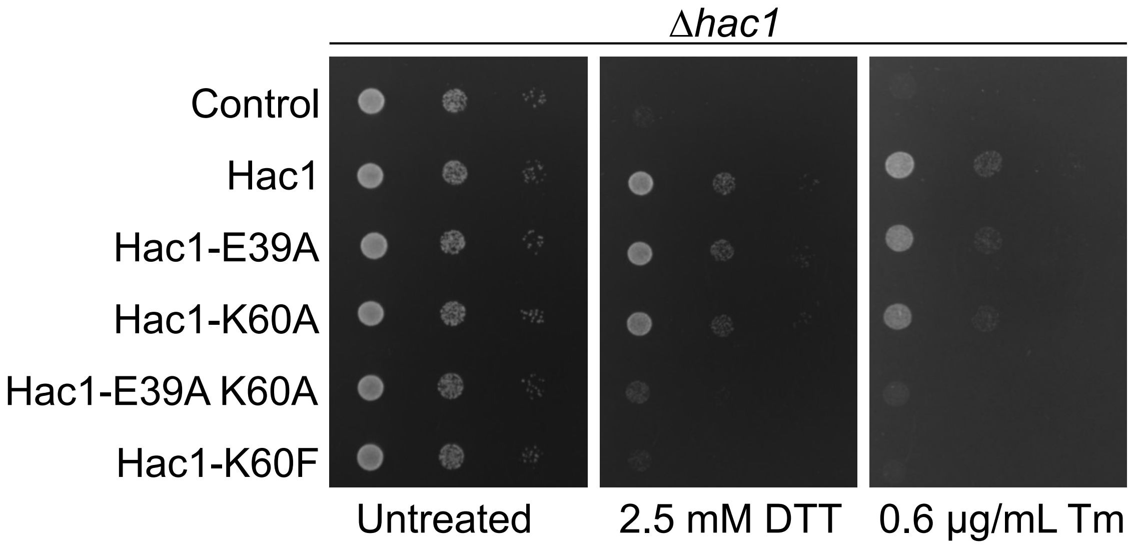

Fig. S12. Sensitivity of Hac1 variants to chemical inducers. The *Δhac1* strains, transformed with specified Hac1 variants, were spotted onto plates supplemented with either DTT or Tm.

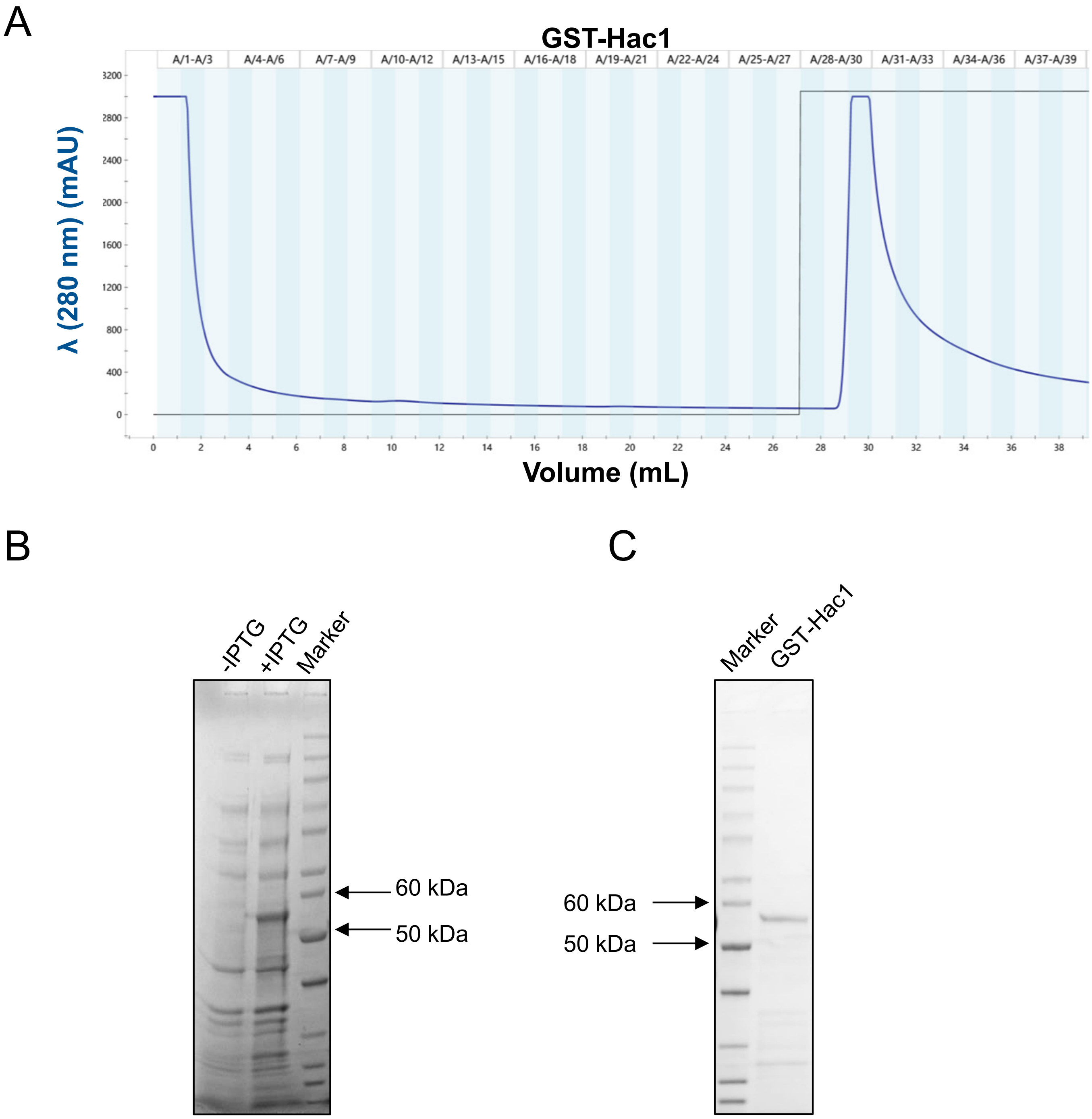

Fig. S13. (A) Fusion proteins GST-Hac1 (52 kDa), efficiently expressed in *E. coli* upon IPTG induction, were purified using glutathione sepharose affinity chromatography resin. (B) SDS-PAGE analysis comparing uninduced and IPTG-induced *E. coli* BL21 (DE3) samples. (C)SDS-PAGE analysis of purified GST-Hac1proteins.

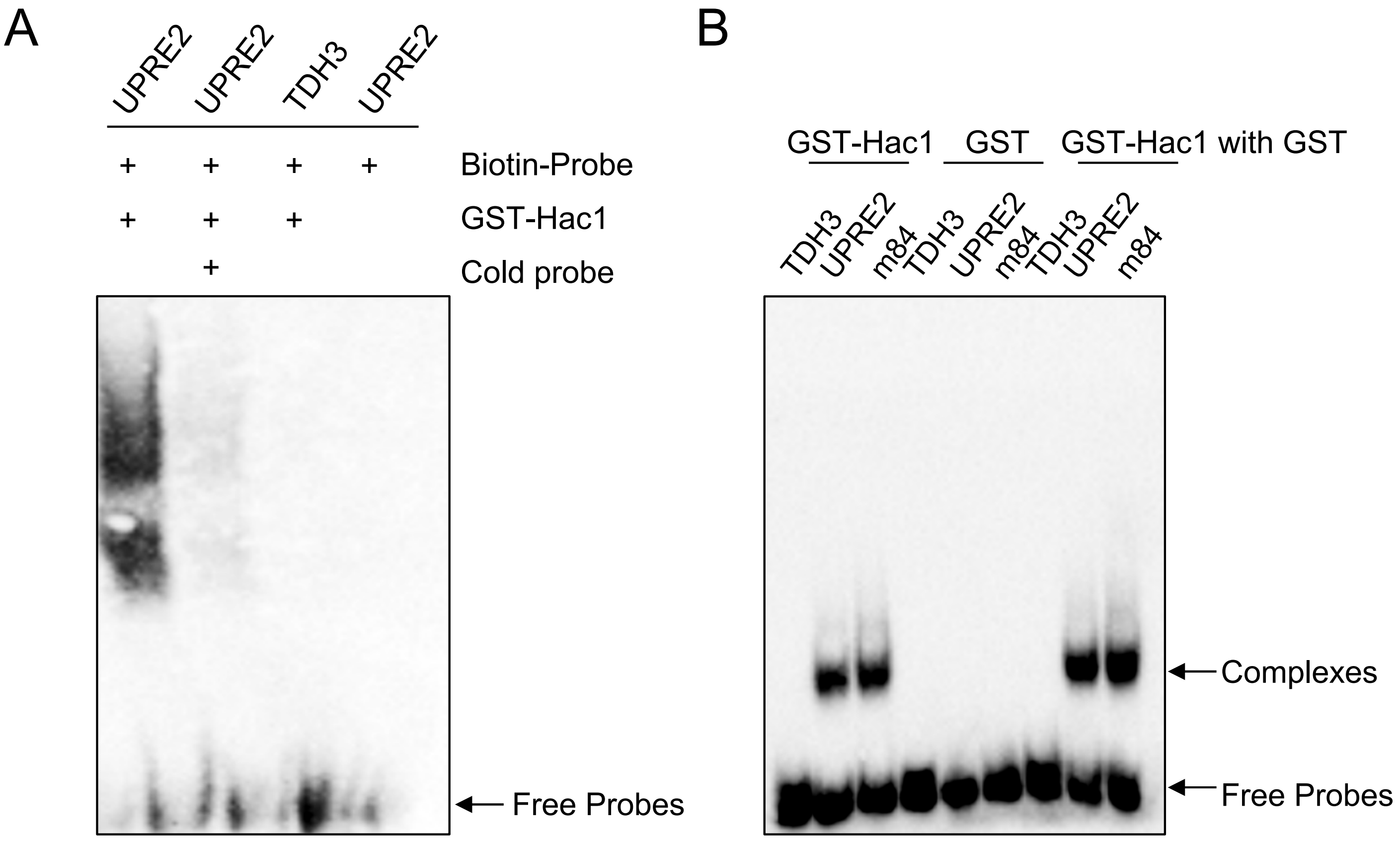

Fig. S14. Specific binding of Hac1 to UPRE2m. (A)The binding of GST-Hac1 to the UPRE2 was assessed using EMSA. (B) The impact of the GST protein on the interaction between GST-Hac1 and UPRE2m.

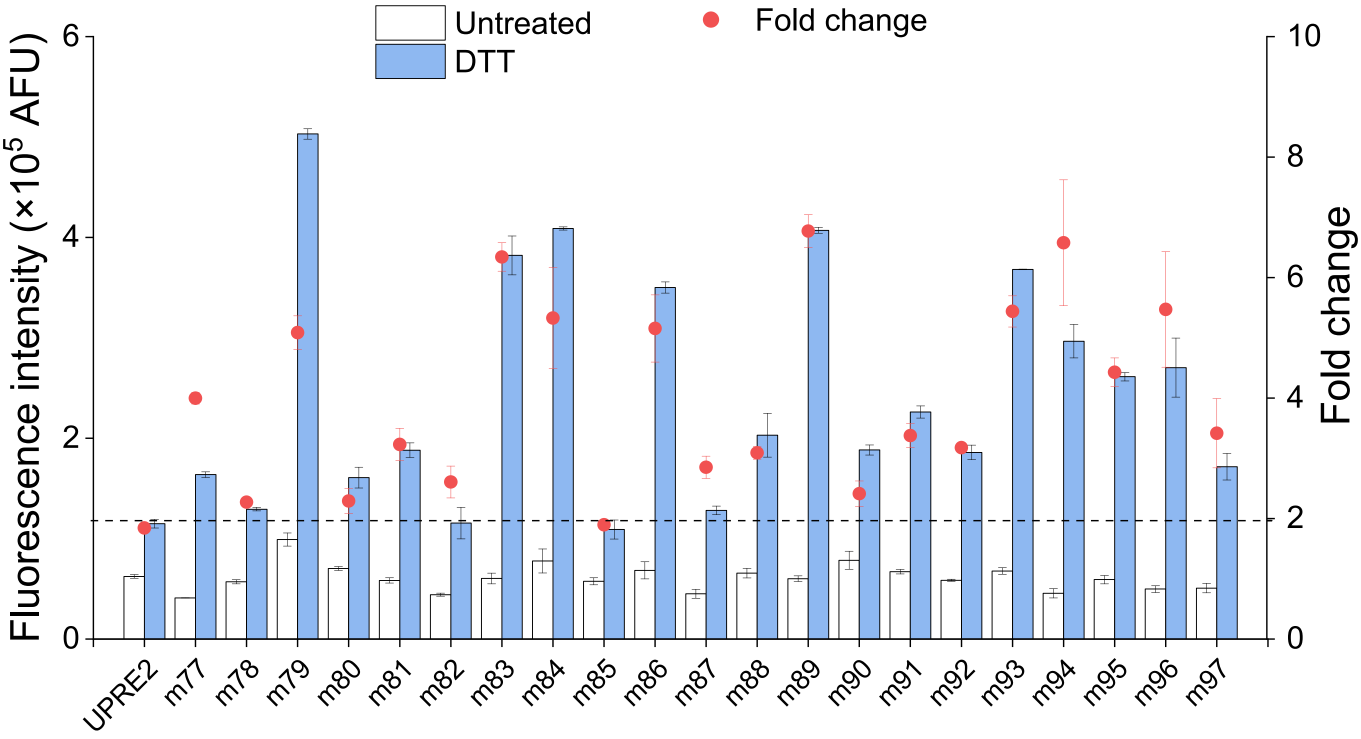

Fig. S15. Response of hybrid promoter UPRE2m-*TDH3*p CORE to 5mM DTT in the BY4742 strain. Data shown are mean values ±SDs of biological duplicates of single clones.

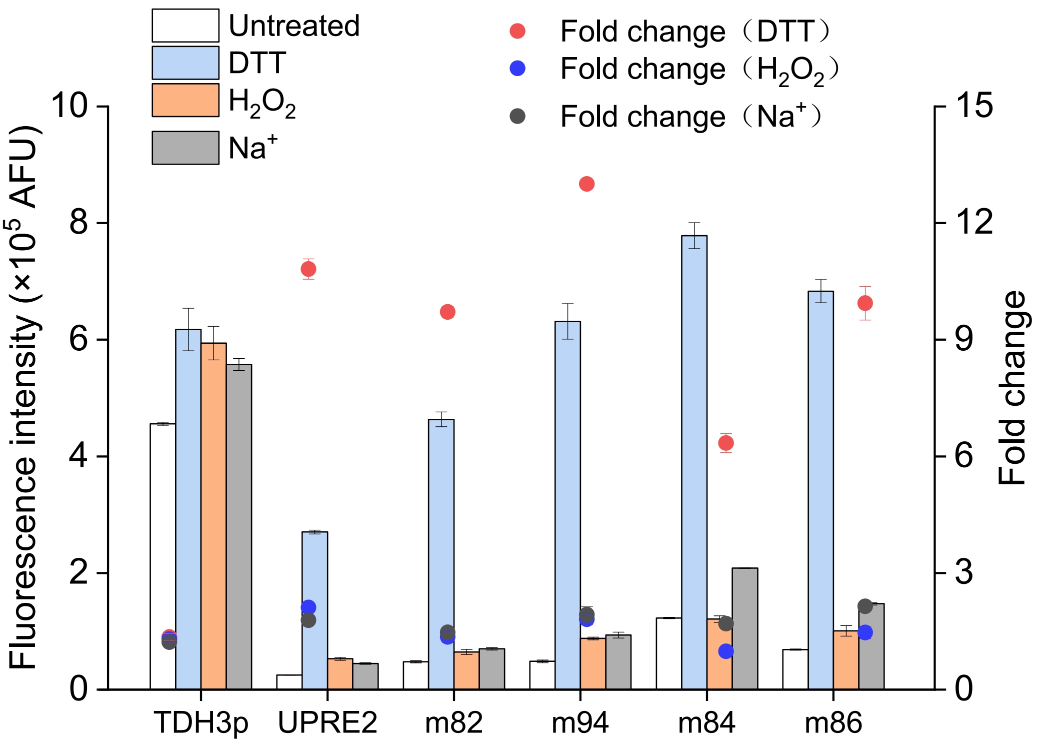

Fig. S16. Response of the hybrid promoter UPRE2m-*TDH3*p CORE to different chemical reagents (5 mM DTT, 0.5 mM H_2O_2, 200 mM NaCl). Data shown are mean values ±SDs of biological duplicates of single clones.

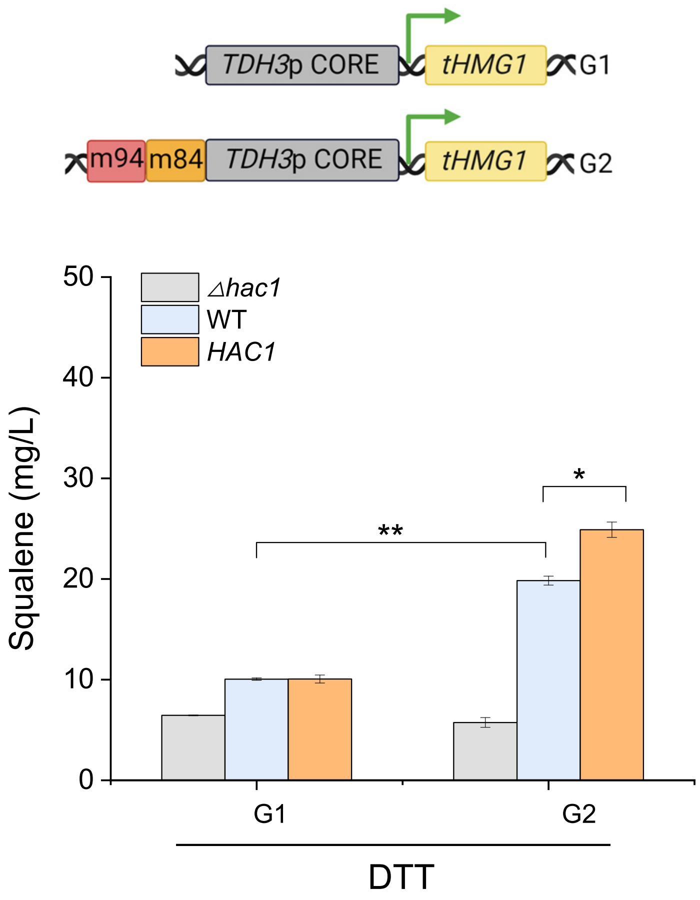

Fig. S17. Effect of promoters (G1: *TDH3*p CORE, G2: m94-m84-*TDH3*p CORE) on regulating *tHMG1* expression and subsequent squalene production. Strains were cultured in YPD medium supplemented with 2.5mM DTT. Data shown are mean values ±SDs of biological duplicates of single clones.

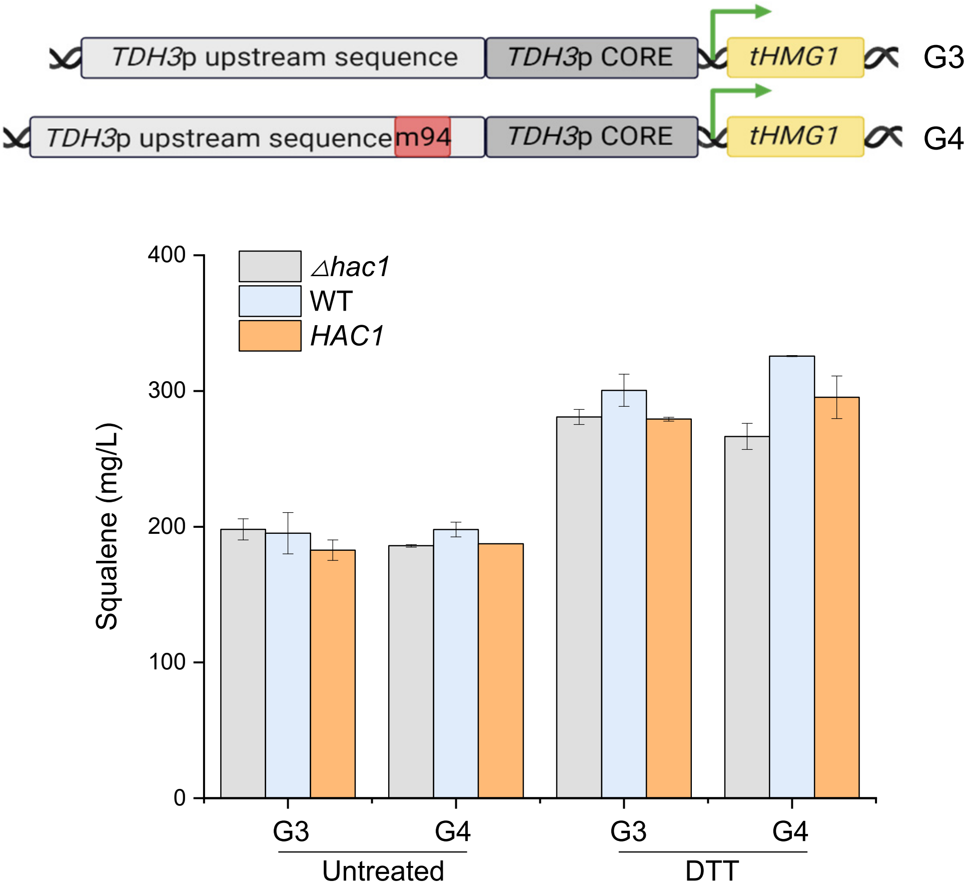

Fig. S18. Impact of the constitutive promoter *TDH3*p (G3) and the responsive hybrid promoter (G4: m94 positioned at the -50 site upstream of *TDH3*p CORE within *TDH3*p) on regulating of *tHMG1* expression and subsequent squalene production in *Δhac1*, WT and *HAC1* overexpression strains. Strains were cultured in YPD medium and YPD medium supplemented with 2.5mM DTT, respectively. Data shown are mean values ±SDs of biological duplicates of single clones.

Table S1 UPRE2m sequence

UPRE2m	Sequence (5'→3')
**Low**	
m1	GAAAGGGGCACGTGTCCGACCAAG
m2	GGGAGTGGTACGTGTCCTCCCTGC
m3	TGCTCCCGTACGTGTCATACATAA
m4	CGCGCCCGCACGTGTCAGCAATAG
m5	ACTGGAGTTACGTGTCCAAAATGC
m6	GTTCGGGTCACGTGTCCGCCCAAG
m7	GGTGGTAGTACGTGTCCGACGAGG
m8	TAGAGCTGCACGTGTCACACGAAA
m9	GGATGGTTCACGTGTCCAACTAGG
m10	CCTTCCATCACGTGTCCTCCGTGG
m11	CGTGGGGGCACGTGTCCTCAGAAT
m12	GGGCGGAGGACGTGTCCCACGAGG
m13	GATCCCGGCACGTGTCCGCAAAGA
m14	GGGGGGTGTACGTGTCACCCGTGT
m15	CCGGGTGGTACGTGTCATACATGA
m16	CCCGGGGTCACGTGTCAACATAGC
m17	CACCGACTCACGTGTCCAACCAAC
m18	GGCGGGCGTACGTGTCAGACGTGG
m19	TCGGGGCTCACGTGTCCGACATGG
m20	GGCCGTGGCACGTGTCCGACCTGG
m21	CGGGCTATCACGTGTCCTACATGC
m22	GGGGGTAGTACGTGTCCCCCCAAA
m23	TTAGCGCGCACGTGTCCGCAGTAA
m24	TTCGGCATCACGTGTCCCCATTGA
m25	AGAGGTTTTACGTGTCACAAGTGG
m26	CCTGGCGGTACGTGTCCTAAGAGC
m27	GGGGGGGGTACGTGTCCCAAATGG
m28	GTGCGCGTCACGTGTCAAACCAGT
m29	CGCGCGTTTACGTGTCCAACGAGG
m30	CGGGCTCGTACGTGTCCCAACTAG
m31	CGTACGGGTACGTGTCAGAATTAG
m32	CGGGCTTGTACGTGTCCTACCTGG
m33	ATGCGGGTTACGTGTCAGAATTGT
m34	TCGGGGGGTACGTGTCCCACATAG
m35	CCTAGTTGTACGTGTCACCCGTAC
**Medium**	
UPRE2	ATACGGAGTACGTGTCATAAAAAC
m36	TGGCCCCTTACGTGTCCCCCTAGG
m37	ATGGGCGTTACGTGTCATCCCTAC
m38	CGGGGGTGTACGTGTCAACACAGG
m39	TTATGCTTCACGTGTCCGAAAAGA

m40	CTCAGGAGCACGTGTCAAGATTGT
m41	CATGGGTTCACGTGTCACACATGC
m42	TCGGGGGGTACGTGTCACCAGAGC
m43	AAGTCCAGCACGTGTCAGCCGAGA
m44	TTTACTTGCACGTGTCACCATTGC
m45	GACGGTTGTACGTGTCAGCACAGC
m46	CAGACCCTCACGTGTCCGAACAGC
m47	TAGTGGTTCACGTGTCATAATAAG
m48	AGAACGCTCACGTGTCCACATAGG
m49	GACGTTGCAACGTGTCCACCGTGG
m50	GAACGGGGTACGTGTCATAACTAG
m51	TCCTGCCTTACGTGTCCACAATGG
m52	GAGTCGGGTACGTGTCCGCAGAAG
m53	CAGGGTTGTACGTGTCCAAAGAGA
m54	TTTCGTGTTACGTGTCCGCCGAGG
m56	GGACGGTTTACGTGTCCGCAAAGA
m57	GTGTGGTGTACGTGTCAACAGAGC
m58	GAGAGAGTTACGTGTCAACAGAGG
m59	AGGGGAATCACGTGTCCTCCATGT
m60	GCCTGACTTACGTGTCCTCAGAGG
m61	CAGTCCGGTACGTGTCCAAAGTAA
m62	TAGGCGTTTACGTGTCATACGTGT
m63	GAGCGAGGTACGTGTCACCAAAAT
m64	CAATGGATTACGTGTCAGCACTAT
m65	TCCACGCTTACGTGTCCTCACTAC
m66	CAGTGTTGTACGTGTCCTCCAAGG
m67	CAAGCGTTCACGTGTCCGAATAGG
**High**	
m72	CAGTGGGTTACGTGTCCGCCATGTC
m73	TAGTCGAGTACGTGTCCAAAGTGA
m74	TGGGCAGTTACGTGTCCTCAAAAA
m75	TGATGGCTTACGTGTCCTCCATGG
m76	GAGCGGTGTACGTGTCCGAAAAG
m77	GAGTCTCTTACGTGTCATAATTAA
m78	GTGTGGGTTACGTGTCCTCCTAAA
m79	CAATGGGGTACGTGTCCTCAAAAT
m80	GGACGGATTACGTGTCCTCAGTGG
m81	GAACGGAGTACGTGTCCTCAAAAC
m82	TTTACGGGTACGTGTCCTCAAAAG
m83	CAGTCAAGTACGTGTCCACATTGT
m84	AAAAGGATTACGTGTCCTCAAAAT
m85	CAGTCGAGTACGTGTCATCATTAC

m86	CAGTGGGTTACGTGTCATCAGAGC
m87	GTGTGGATTACGTGTCCTCACTGG
m88	GTCTCGCTTACGTGTCCTCAAAAA
m89	CAGCGGATTACGTGTCCCACAAGC
m90	GAAAGCGTTACGTGTCCAAAAAGA
m91	GAGTGAGTTACGTGTCCAAAGAGA
m92	GAGCGTGTTACGTGTCATCACAGC
m93	AAGCGCGTTACGTGTCCCCAAAAT
m94	AAGCGGTTTACGTGTCAAAAGTGT
m95	CTGCGGTTTACGTGTCCACAAAGC
m96	CAGTCGCTTACGTGTCCTACTAGT
m97	TTAGCATGTACGTGTCATAAATGA

Table S2 The EC_{50} of strains containing UPRE2m-*TDH3*p CORE.

**UPRE2m**	**DTT EC_{50} (mM)**	**Tm EC_{50} (μg/mL)**
UPRE2	1.79561	0.31724
m87	1.52662	0.29344
m84	12.13073	0.33386
m86	1.6519	0.30866
m94	1.95418	0.28736
m82	1.93249	0.36273
m77	1.74425	0.33724

- EC_{50} defined as the concentration that leads to 50% of the maximum effect.
- Since the response of component m84 is not saturated at the highest DTT dose, its predicted EC_{50} may be biased, exceeding the highest DTT dose used in this study.

Table S3 Plasmids used in this study.

Plasmids	Genotype	Source
pUC57-UPRE2-*TDH3*p-EforRed	pUC57-(UPRE2-*TDH3*p-EforRed-*CYC1*t)	GenScript Co.
p426GPD	2 μm, AmpR, *URA3*, *GPD*p, *CYC1*t	(1)
p416GPD	CEN6/ARSH4, AmpR, *URA3*, *GPD*p, *CYC1*t	(1)
pUC57-Mini-RFP-GFP	pUC57-Mini-(RFP-GFP)	GenScript Co.
pROS10	2μm,AmpR, *URA3*, *SNR52*p, gRNA scaffold, *SUP4* t, *SNR52*p, gRNA scaffold, *SUP4*t	EUROSCARF
pROS10-X-3	pROS10-(*SNR52*p-X-3-scaffold-*SUP4*t)	This study
pROS10-XII-5	pROS10-(*SNR52*p-XII-5-scaffold-*SUP4*t)	This study
pROS10-X-3-XII-2	pROS10-(*SNR52*p-X-3-scaffold-*SUP4*t-*SNR52*p-XII-2-scaffold-*SUP4*t)	This study
p426-UPRE2-*TDH3*p-EforRed-GFP	p426-(UPRE2-*TDH3*p CORE-EforRed-GFP-*CYC1*t)	This study
p416-UPRE2-*TDH3*p-EforRed-GFP	p416-(UPRE2-*TDH3*p CORE -EforRed-GFP-*CYC1*t)	This study
p426-*TDH3*p-EforRed-GFP	p426-(*TDH3*p-EforRed-GFP-*CYC1*t)	This study
p426-*TEF1*p-EforRed-GFP	p426-(*TEF1*p-EforRed-GFP-*CYC1*t)	This study
p426-*TPI1*p-EforRed-GFP	p426-(*TPI1*p-EforRed-GFP-*CYC1*t)	This study
p426-*CYC1*p-EforRed-GFP	p426-(*CYC1*p -EforRed-GFP-*CYC1*t)	This study
p416-*TDH3*p-EforRed-GFP	p416-(*CYC1*p-EforRed-GFP-*CYC1*t)	This study
p416-*TEF1*p-EforRed-GFP	p416-(*TEF1*p-EforRed-GFP-*CYC1*t)	This study
p416-*TPI1*p-EforRed-GFP	p416-(*TPI1*p-EforRed-GFP-*CYC1*t)	This study
p416-*CYC1*p-EforRed-GFP	p416-(*CYC1*p-EforRed-GFP-*CYC1*t)	This study
p416-m94-m84-*PDC1*p-RFP	p416-(m94-m84-*PDC1*p CORE-RFP-*CYC1*t)	This study
p416-m94-m84-*PGK1*p-RFP	p416-(m94-m84-*PGK1*p CORE-RFP-*CYC1*t)	This study
p416-m94-m84-*TDH3*p-RFP	p416-(m94-m84-*TDH3*p CORE-RFP-*CYC1*t)	This study
p416-m94-m84-*TEF1*p-RFP	p416-(m94-m84-*TEF1*p CORE-RFP-*CYC1*t)	This study
p416-m94-m84-*TPI1*p-RFP	p416-(m94-m84-*TPI1*p CORE-RFP-*CYC1*t)	This study
pCas	2μm, AmpR, *TEF1*p-iCas9 *SNR52*p	(2)
psgtRNA	ori, AmpR, gRNA-t*Gly*	(2)
pScURA	ori, AmpR, gRNA-*URA3*-*SNR52*p-t*Gly*	(2)
pCas-X-3	pCas with double gRNA-X-3	This study
pCP-*TPI1*p-Aamylase	CPOTUd-(*TPI1*p-Aamylase-*TPI1*t)	This study
pls-14-pY26-m94-*TDH3*p- t*HMG1*	pls-14-pY26-(m94-*TDH3*p -t*HMG1*-*CYC1*t)	This study
pls-14-pY26-*TDH3*p- t*HMG1*	pls-14-pY26-(*TDH3*p-t*HMG1*-*CYC1*t)	This study
pls-14-pY26-m94-m84-*TDH3*p CORE-t*HMG1*	pls-14-pY26-(m94-m84-*TDH3*p CORE -t*HMG1*-*CYC1*t)	This study
pGEX-GST-Hac1	pGEX -(T7p- GST-*HAC1*-T7t)	This study

Table S4 Yeast strains used in this study.

Strain	Genotype	Source
IMX581	*MATa ura3-52 can1_::cas9-natNT2 TRP1 LEU2 HIS3*	EUROSCARF
CF1001	IMX581 / p426-UPRE2-*TDH3*p CORE-EforRed-GFP	This study
CF1002	IMX581 / p416-UPRE2-*TDH3*p CORE-EforRed-GFP	This study
CF1003	IMX581 X-3::UPRE2-*TDH3*p CORE-EforRed-GFP	This study
CF1004	IMX581 / p426-*TDH3*p-EforRed-GFP	This study
CF1005	IMX581 / p426-*TEF1*p-EforRed-GFP	This study
CF1006	IMX581 / p426-*TPI1*p-EforRed-GFP	This study
CF1007	IMX581 / p426-*CYC1*p-EforRed-GFP	This study
CF1008	IMX581 / p416-*TDH3*p-EforRed-GFP	This study
CF1009	IMX581 / p416-*TEF1*p-EforRed-GFP	This study
CF1010	IMX581 / p416-*TPI1*p-EforRed-GFP	This study
CF1011	IMX581 / p416-*CYC1*p-EforRed-GFP	This study
CF1012	IMX581 X-3:: UPRE2-m84-*TDH3*p CORE-RFP-*CYC1*t	This study
CF1013	IMX581 X-3:: UPRE2-m86-*TDH3*p CORE-RFP-*CYC1*t	This study
CF1014	IMX581 X-3:: m94-m84-*TDH3*p CORE-RFP-*CYC1*t	This study
CF1015	IMX581 X-3:: m94-m86-*TDH3*p CORE-RFP-*CYC1*t	This study
CF1016	IMX581 X-3:: m94-m94-*TDH3*p CORE-RFP-*CYC1*t	This study
CF1017	IMX581 X-3:: UPRE2-UPRE2-*TDH3*p CORE-RFP-*CYC1*t	This study
CF1018	IMX581 X-3:: m84-m84-*TDH3*p CORE-RFP-*CYC1*t	This study
CF1019	IMX581 X-3:: m86-m86-*TDH3*p CORE-RFP-*CYC1*t	This study
CF1020	IMX581 X-3:: m94-UPRE2-*TDH3*p CORE-RFP-*CYC1*t	This study
CF1021	IMX581 X-3:: UPRE2-m94-*TDH3*p CORE-RFP-*CYC1*t	This study
CF1022	IMX581 /p416-m94-m84-*PDC1*p CORE-RFP	This study
CF1023	IMX581 /p416-m94-m84-*PGK1*p CORE-RFP	This study
CF1024	IMX581 /p416-m94-m84-*TDH3*p CORE-RFP	This study
CF1025	IMX581 /p416-m94-m84-*TEF1*p CORE-RFP	This study
CF1026	IMX581 /p416-m94-m84-*TPI1*p CORE-RFP	This study
CF1027	IMX581 / p416-UPRE2-*TDH3*p CORE-RFP	This study
CF1028	IMX581 Δ*hac1* X-3::UPRE2-*TDH3*p CORE-RFP-*CYC1*t	This study
CF1029	IMX581 Δ*hac1* X-3::m82-*TDH3*p CORE-RFP-*CYC1*t	This study
CF1030	IMX581 Δ*hac1* X-3::m84-*TDH3*p CORE-RFP-*CYC1*t	This study
CF1031	IMX581 Δ*hac1* X-3::m94-*TDH3*p CORE-RFP-*CYC1*t	This study
CF1032	IMX581 Δ*hac1* X-3::m86-*TDH3*p CORE-RFP-*CYC1*t	This study
CF1033	IMX581 Δ*hac1* X-3::*TDH3*p -RFP-*CYC1*t	This study
CF1034	IMX581 Δ*gcn4* X-3::UPRE2-*TDH3*p CORE-RFP-*CYC1*t	This study
CF1035	IMX581 Δ*gcn4* X-3::m82-*TDH3*p CORE-RFP-*CYC1*t	This study
CF1036	IMX581 Δ*gcn4* X-3::m84-*TDH3*p CORE-RFP-*CYC1*t	This study
CF1037	IMX581 Δ*gcn4* X-3::m94-*TDH3*p CORE-RFP-*CYC1*t	This study
CF1038	IMX581 Δ*gcn4* X-3::m86-*TDH3*p CORE-RFP-*CYC1*t	This study
CF1039	IMX581 Δ*gcn4* X-3::*TDH3*p-RFP-*CYC1*t	This study
CF1040	IMX581 Δ*hac1*Δ*gcn4* X-3::UPRE2-*TDH3*p CORE-RFP-*CYC1*t	This study
CF1041	IMX581 Δ*hac1*Δ*gcn4* X-3::m82-*TDH3*p CORE-RFP-*CYC1*t	This study
CF1042	IMX581 Δ*hac1*Δ*gcn4* X-3::m84-*TDH3*p CORE-RFP-*CYC1*t	This study

CF1043	IMX581 Δ*hac1*Δ*gcn4* X-3::m94-*TDH3*p CORE-RFP-*CYC1*t	This study
CF1044	IMX581 Δ*hac1*Δ*gcn4* X-3::m86-*TDH3*p CORE-RFP-*CYC1*t	This study
CF1045	IMX581 Δ*hac1*Δ*gcn4* X-3::*TDH3*p-RFP-*CYC1*t	This study
CF1046	IMX581 Δ*haa1* X-3::UPRE2-*TDH3*p CORE-RFP-*CYC1*t	This study
CF1047	IMX581 Δ*haa1* X-3::m82-*TDH3*p CORE-RFP-*CYC1*t	This study
CF1048	IMX581 Δ*haa1* X-3::m84-*TDH3*p CORE-RFP-*CYC1*t	This study
CF1049	IMX581 Δ*haa1* X-3::m94-*TDH3*p CORE-RFP-*CYC1*t	This study
CF1050	IMX581 Δ*haa1* X-3::m86-*TDH3*p CORE-RFP-*CYC1*t	This study
CF1051	IMX581 Δ*haa1* X-3::*TDH3*p-RFP-*CYC1*t	This study
CF1052	IMX581 Δ*hac1* XII-5::*HAC1* X-3::UPRE2-*TDH3*p CORE-RFP-*CYC1*t	This study
CF1053	IMX581 Δ*hac1* XII-5::*HAC1* X-3::m82-*TDH3*p CORE-RFP-*CYC1*t	This study
CF1054	IMX581 Δ*hac1* XII-5::*HAC1* X-3::m84-*TDH3*p CORE-RFP-*CYC1*t	This study
CF1054	IMX581 Δ*hac1* XII-5::*HAC1* X-3::m94-*TDH3*p CORE-RFP-*CYC1*t	This study
CF1055	IMX581 Δ*hac1* XII-5::*HAC1* X-3::m86-*TDH3*p CORE-RFP-*CYC1*t	This study
CF1056	IMX581 Δ*hac1* XII-5::*HAC1-E39A* X-3::UPRE2-*TDH3*p CORE-RFP-*CYC1*t	This study
CF1057	IMX581 Δ*hac1* XII-5::*HAC1-E39A* X-3::m82-*TDH3*p CORE-RFP-*CYC1*t	This study
CF1058	IMX581 Δ*hac1* XII-5::*HAC1-E39A* X-3::m84-*TDH3*p CORE-RFP-*CYC1*t	This study
CF1059	IMX581 Δ*hac1* XII-5::*HAC1-E39A* X-3::m94-*TDH3*p CORE-RFP-*CYC1*t	This study
CF1060	IMX581 Δ*hac1* XII-5::*HAC1-E39A* X-3::m86-*TDH3*p CORE-RFP-*CYC1*t	This study
CF1061	IMX581 Δ*hac1* XII-5::*HAC1-K60A* X-3::UPRE2-*TDH3*p CORE -RFP-*CYC1*t	This study
CF1062	IMX581 Δ*hac1* XII-5::*HAC1-K60A* X-3::m82-*TDH3*p CORE-RFP-*CYC1*t	This study
CF1063	IMX581 Δ*hac1* XII-5::*HAC1-K60A* X-3::m84-*TDH3*p CORE-RFP-*CYC1*t	This study
CF1064	IMX581 Δ*hac1* XII-5::*HAC1-K60A* X-3::m94-*TDH3*p CORE-RFP-*CYC1*t	This study
CF1065	IMX581 Δ*hac1* XII-5::*HAC1-K60A* X-3::m86-*TDH3*p CORE-RFP-*CYC1*t	This study
CF1066	IMX581 Δ*hac1* XII-5::*HAC1-E39A K60A* X-3::UPRE2-*TDH3*p CORE -RFP-*CYC1*t	This study
CF1067	IMX581 Δ*hac1* XII-5::*HAC1-E39A K60A* X-3::m82-*TDH3*p CORE-RFP-*CYC1*t	This study
CF1068	IMX581 Δ*hac1* XII-5::*HAC1- E39A K60A* X-3::m84-*TDH3*p CORE-RFP-*CYC1*t	This study
CF1069	IMX581 Δ*hac1* XII-5::*HAC1- E39A K60A* X-3::m94-*TDH3*p CORE-RFP-*CYC1*t	This study

CF1070	IMX581 Δ*hac1* XII-5::*HAC1- E39A K60A* X-3::m86-*TDH3*p CORE-RFP-*CYC1*t	This study
CF1071	IMX581 Δ*hac1* XII-5::*HAC1-K60E* X-3::UPRE2-*TDH3*p CORE-RFP-*CYC1*t	This study
CF1072	IMX581 Δ*hac1* XII-5::*HAC1-K60E* X-3::m82-*TDH3*p CORE-RFP-*CYC1*t	This study
CF1073	IMX581 Δ*hac1* XII-5::*HAC1-K60E* X-3::m84-*TDH3*p CORE-RFP-*CYC1*t	This study
CF1074	IMX581 Δ*hac1* XII-5::*HAC1-K60E* X-3::m94-*TDH3*p CORE-RFP-*CYC1*t	This study
CF1075	IMX581 Δ*hac1* XII-5::*HAC1-K60E* X-3::m86-*TDH3*p CORE-RFP-*CYC1*t	This study
CF1076	IMX581 Δ*hac1* XII-5::*HAC1-K60M* X-3::UPRE2-*TDH3*p CORE-RFP-*CYC1*t	This study
CF1077	IMX581 Δ*hac1* XII-5::*HAC1-K60M* X-3::m82-*TDH3*p CORE-RFP-*CYC1*t	This study
CF1078	IMX581 Δ*hac1* XII-5::*HAC1-K60M* X-3::m84-*TDH3*p CORE-RFP-*CYC1*t	This study
CF1079	IMX581 Δ*hac1* XII-5::*HAC1-K60M* X-3::m94-*TDH3*p CORE-RFP-*CYC1*t	This study
CF1080	IMX581 Δ*hac1* XII-5::*HAC1-K60M* X-3::m86-*TDH3*p CORE-RFP-*CYC1*t	This study
CF1081	IMX581 Δ*hac1* XII-5::*HAC1-K60H* X-3::UPRE2-*TDH3*p CORE-RFP-*CYC1*t	This study
CF1082	IMX581 Δ*hac1* XII-5::*HAC1-K60H* X-3::m82-*TDH3*p CORE-RFP-*CYC1*t	This study
CF1083	IMX581 Δ*hac1* XII-5::*HAC1-K60H* X-3::m84-*TDH3*p CORE-RFP-*CYC1*t	This study
CF1084	IMX581 Δ*hac1* XII-5::*HAC1-K60H* X-3::m94-*TDH3*p CORE-RFP-*CYC1*t	This study
CF1085	IMX581 Δ*hac1* XII-5::*HAC1-K60H* X-3::m86-*TDH3*p CORE-RFP-*CYC1*t	This study
CF1086	IMX581 Δ*hac1* XII-5::*HAC1-K60C* X-3::UPRE2-*TDH3*p CORE-RFP-*CYC1*t	This study
CF1087	IMX581 Δ*hac1* XII-5::*HAC1-K60C* X-3::m82-*TDH3*p CORE-RFP-*CYC1*t	This study
CF1088	IMX581 Δ*hac1* XII-5::*HAC1-K60C* X-3::m84-*TDH3*p CORE-RFP-*CYC1*t	This study
CF1089	IMX581 Δ*hac1* XII-5::*HAC1-K60C* X-3::m94-*TDH3*p CORE-RFP-*CYC1*t	This study
CF1090	IMX581 Δ*hac1* XII-5::*HAC1-K60C* X-3::m86-*TDH3*p CORE-RFP-*CYC1*t	This study
CF1091	IMX581 Δ*hac1* XII-5::*HAC1-K60L* X-3::UPRE2-*TDH3*p CORE-RFP-*CYC1*t	This study
CF1092	IMX581 Δ*hac1* XII-5::*HAC1-K60L* X-3::m82-*TDH3*p CORE-RFP-*CYC1*t	This study
CF1093	IMX581 Δ*hac1* XII-5::*HAC1-K60L*	This study

	X-3::m84-*TDH3*p CORE-RFP-*CYC1*t	
CF1094	IMX581 Δ*hac1* XII-5::*HAC1-K60L* X-3::m94-*TDH3*p CORE-RFP-*CYC1*t	This study
CF1095	IMX581 Δ*hac1* XII-5::*HAC1-K60L* X-3::m86-*TDH3*p CORE-RFP-*CYC1*t	This study
CF1096	IMX581 Δ*hac1* XII-5::*HAC1-K60G* X-3::UPRE2-*TDH3*p CORE-RFP-*CYC1*t	This study
CF1097	IMX581 Δ*hac1* XII-5::*HAC1-K60G* X-3::m82-*TDH3*p CORE-RFP-*CYC1*t	This study
CF1098	IMX581 Δ*hac1* XII-5::*HAC1-K60G* X-3::m84-*TDH3*p CORE-RFP-*CYC1*t	This study
CF1099	IMX581 Δ*hac1* XII-5::*HAC1-K60G* X-3::m94-*TDH3*p CORE-RFP-*CYC1*t	This study
CF1100	IMX581 Δ*hac1* XII-5::*HAC1-K60G* X-3::m86-*TDH3*p CORE-RFP-*CYC1*t	This study
CF1101	IMX581 Δ*hac1* XII-5::*HAC1-K60R* X-3::UPRE2-*TDH3*p CORE-RFP-*CYC1*t	This study
CF1102	IMX581 Δ*hac1* XII-5::*HAC1-K60R* X-3::m82-*TDH3*p CORE-RFP-*CYC1*t	This study
CF1103	IMX581 Δ*hac1* XII-5::*HAC1-K60R* X-3::m84-*TDH3*p CORE-RFP-*CYC1*t	This study
CF1104	IMX581 Δ*hac1* XII-5::*HAC1-K60R* X-3::m94-*TDH3*p CORE-RFP-*CYC1*t	This study
CF1105	IMX581 Δ*hac1* XII-5::*HAC1-K60R* X-3::m86-*TDH3*p CORE-RFP-*CYC1*t	This study
CF1106	IMX581 Δ*hac1* XII-5::*HAC1-K60F* X-3::UPRE2-*TDH3*p CORE-RFP-*CYC1*t	This study
CF1107	IMX581 Δ*hac1* XII-5::*HAC1-K60F* X-3::m82-*TDH3*p CORE-RFP-*CYC1*t	This study
CF1108	IMX581 Δ*hac1* XII-5::*HAC1-K60F* X-3::m84-*TDH3*p CORE-RFP-*CYC1*t	This study
CF1109	IMX581 Δ*hac1* XII-5::*HAC1-K60F* X-3::m94-*TDH3*p CORE-RFP-*CYC1*t	This study
CF1110	IMX581 Δ*hac1* XII-5::*HAC1-K60F* X-3::m86-*TDH3*p CORE-RFP-*CYC1*t	This study
CF1111	IMX581 Δ*hac1* XII-5::*HAC1-K60D* X-3::UPRE2-*TDH3*p CORE-RFP-*CYC1*t	This study
CF1112	IMX581 Δ*hac1* XII-5::*HAC1-K60D* X-3::m82-*TDH3*p CORE-RFP-*CYC1*t	This study
CF1113	IMX581 Δ*hac1* XII-5::*HAC1-K60D* X-3::m84-*TDH3*p CORE-RFP-*CYC1*t	This study
CF1114	IMX581 Δ*hac1* XII-5::*HAC1-K60D* X-3::m94-*TDH3*p CORE-RFP-*CYC1*t	This study
CF1115	IMX581 Δ*hac1* XII-5::*HAC1-K60D* X-3::m86-*TDH3*p CORE-RFP-*CYC1*t	This study
CF1116	IMX581 Δ*hac1* XII-5::*HAC1-K60S* X-3::UPRE2-*TDH3*p CORE-RFP-*CYC1*t	This study

CF1117	IMX581 Δ*hac1* XII-5::*HAC1-K60S* X-3::m82-*TDH3*p CORE-RFP-*CYC1*t	This study
CF1118	IMX581 Δ*hac1* XII-5::*HAC1-K60S* X-3::m84-*TDH3*p CORE-RFP-*CYC1*t	This study
CF1119	IMX581 Δ*hac1* XII-5::*HAC1-K60S* X-3::m94-*TDH3*p CORE-RFP-*CYC1*t	This study
CF1120	IMX581 Δ*hac1* XII-5::*HAC1-K60S* X-3::m86-*TDH3*p CORE-RFP-*CYC1*t	This study
CF1121	IMX581 Δ*hac1* XII-5::*HAC1-K60W* X-3::UPRE2-*TDH3*p CORE-RFP-*CYC1*t	This study
CF1122	IMX581 Δ*hac1* XII-5::*HAC1-K60W* X-3::m82-*TDH3*p CORE-RFP-*CYC1*t	This study
CF1123	IMX581 Δ*hac1* XII-5::*HAC1-K60W* X-3::m84-*TDH3*p CORE-RFP-*CYC1*t	This study
CF1124	IMX581 Δ*hac1* XII-5::*HAC1-K60W* X-3::m94-*TDH3*p CORE-RFP-*CYC1*t	This study
CF1125	IMX581 Δ*hac1* XII-5::*HAC1-K60W* X-3::m86-*TDH3*p CORE-RFP-*CYC1*t	This study
CF1126	IMX581 Δ*hac1* XII-5::*HAC1-K60Q* X-3::UPRE2-*TDH3*p CORE-RFP-*CYC1*t	This study
CF1127	IMX581 Δ*hac1* XII-5::*HAC1-K60Q* X-3::m82-*TDH3*p CORE-RFP-*CYC1*t	This study
CF1128	IMX581 Δ*hac1* XII-5::*HAC1-K60Q* X-3::m84-*TDH3*p CORE-RFP-*CYC1*t	This study
CF1129	IMX581 Δ*hac1* XII-5::*HAC1-K60Q* X-3::m94-*TDH3*p CORE-RFP-*CYC1*t	This study
CF1130	IMX581 Δ*hac1* XII-5::*HAC1-K60Q* X-3::m86-*TDH3*p CORE-RFP-*CYC1*t	This study
CF1131	IMX581 Δ*hac1* XII-5::*HAC1-K60P* X-3::UPRE2-*TDH3*p CORE-RFP-*CYC1*t	This study
CF1132	IMX581 Δ*hac1* XII-5::*HAC1-K60P* X-3::m82-*TDH3*p CORE-RFP-*CYC1*t	This study
CF1133	IMX581 Δ*hac1* XII-5::*HAC1-K60P* X-3::m84-*TDH3*p CORE-RFP-*CYC1*t	This study
CF1134	IMX581 Δ*hac1* XII-5::*HAC1-K60P* X-3::m94-*TDH3*p CORE-RFP-*CYC1*t	This study
CF1135	IMX581 Δ*hac1* XII-5::*HAC1-K60P* X-3::m86-*TDH3*p CORE-RFP-*CYC1*t	This study
CF1136	IMX581 Δ*hac1* XII-5::*HAC1-K60T* X-3::UPRE2-*TDH3*p CORE-RFP-*CYC1*t	This study
CF1137	IMX581 Δ*hac1* XII-5::*HAC1-K60T* X-3::m82-*TDH3*p CORE-RFP-*CYC1*t	This study
CF1138	IMX581 Δ*hac1* XII-5::*HAC1-K60T* X-3::m84-*TDH3*p CORE-RFP-*CYC1*t	This study
CF1139	IMX581 Δ*hac1* XII-5::*HAC1-K60T* X-3::m94-*TDH3*p CORE-RFP-*CYC1*t	This study
CF1140	IMX581 Δ*hac1* XII-5::*HAC1-K60T*	This study

	X-3::m86-*TDH3*p CORE-RFP-*CYC1*t	
CF1141	IMX581 Δ*hac1* XII-5::*HAC1-K60Y*<br>X-3::UPRE2-*TDH3*p CORE-RFP-*CYC1*t	This study
CF1142	IMX581 Δ*hac1* XII-5::*HAC1-K60Y*<br>X-3::m82-*TDH3*p CORE-RFP-*CYC1*t	This study
CF1143	IMX581 Δ*hac1* XII-5::*HAC1-K60Y*<br>X-3::m84-*TDH3*p CORE-RFP-*CYC1*t	This study
CF1144	IMX581 Δ*hac1* XII-5::*HAC1-K60Y*<br>X-3::m94-*TDH3*p CORE-RFP-*CYC1*t	This study
CF1145	IMX581 Δ*hac1* XII-5::*HAC1-K60Y*<br>X-3::m86-*TDH3*p CORE-RFP-*CYC1*t	This study
CF1146	IMX581 Δ*hac1* XII-5::*HAC1-K60V*<br>X-3::UPRE2-*TDH3*p CORE-RFP-*CYC1*t	This study
CF1147	IMX581 Δ*hac1* XII-5::*HAC1-K60V*<br>X-3::m82-*TDH3*p CORE-RFP-*CYC1*t	This study
CF1148	IMX581 Δ*hac1* XII-5::*HAC1-K60V*<br>X-3::m84-*TDH3*p CORE-RFP-*CYC1*t	This study
CF1149	IMX581 Δ*hac1* XII-5::*HAC1-K60V*<br>X-3::m94-*TDH3*p CORE-RFP-*CYC1*t	This study
CF1150	IMX581 Δ*hac1* XII-5::*HAC1-K60V*<br>X-3::m86-*TDH3*p CORE-RFP-*CYC1*t	This study
CF1151	IMX581 Δ*hac1* XII-5::*HAC1-K60N*<br>X-3::UPRE2-*TDH3*p CORE-RFP-*CYC1*t	This study
CF1152	IMX581 Δ*hac1* XII-5::*HAC1-K60N*<br>X-3::m82-*TDH3*p CORE-RFP-*CYC1*t	This study
CF1153	IMX581 Δ*hac1* XII-5::*HAC1-K60N*<br>X-3::m84-*TDH3*p CORE-RFP-*CYC1*t	This study
CF1154	IMX581 Δ*hac1* XII-5::*HAC1-K60N*<br>X-3::m94-*TDH3*p CORE-RFP-*CYC1*t	This study
CF1155	IMX581 Δ*hac1* XII-5::*HAC1-K60N*<br>X-3::m86-*TDH3*p CORE-RFP-*CYC1*t	This study
CF1156	IMX581 Δ*hac1* XII-5::*HAC1-K60I*<br>X-3::UPRE2-*TDH3*p CORE-RFP-*CYC1*t	This study
CF1157	IMX581 Δ*hac1* XII-5::*HAC1-K60I*<br>X-3::m82-*TDH3*p CORE-RFP-*CYC1*t	This study
CF1158	IMX581 Δ*hac1* XII-5::*HAC1-K60I*<br>X-3::m84-*TDH3*p CORE-RFP-*CYC1*t	This study
CF1159	IMX581 Δ*hac1* XII-5::*HAC1-K60I*<br>X-3::m94-*TDH3*p CORE-RFP-*CYC1*t	This study
CF1160	IMX581 Δ*hac1* XII-5::*HAC1-K60I*<br>X-3::m86-*TDH3*p CORE-RFP-*CYC1*t	This study
CF1162	IMX581 Δ*hac1* XII-5::*HAC1* X-3:: UPRE2-*TDH3*p CORE-RFP-*CYC1*t	This study
CF1163	IMX581 Δ*hac1* XII-5::*HAC1*-2Be<br>X-3:: UPRE2-*TDH3*p CORE-RFP-*CYC1*t	This study
CF1164	IMX581 Δ*hac1* XII-5::*HAC1*-S238D<br>X-3:: UPRE2-*TDH3*p CORE-RFP-*CYC1*t	This study

CF1165	IMX581 Δ*hac1* XII-5::*HAC1*-Med2 X-3:: UPRE2-*TDH3*p CORE-RFP-*CYC1*t	This study
CF1166	IMX581 Δ*hac1* XII-5::*HAC1*-Gal4 X-3:: UPRE2-*TDH3*p CORE-RFP-*CYC1*t	This study
CF1167	IMX581 XII-5::*HAC1* X-3:: UPRE2-*TDH3*p CORE-RFP-*CYC1*t	This study
CF1168	IMX581 XII-5::*HAC1* X-3:: m82-*TDH3*p CORE-RFP-*CYC1*t	This study
CF1169	IMX581 XII-5::*HAC1* X-3:: m84-*TDH3*p CORE-RFP-*CYC1*t	This study
CF1170	IMX581 XII-5::*HAC1* X-3:: m86-*TDH3*p CORE-RFP-*CYC1*t	This study
CF1171	IMX581 XII-5::*HAC1* X-3:: m94-*TDH3*p CORE-RFP-*CYC1*t	This study
CF1172	IMX581 XII-5::*HAC1* X-3:: *TDH3*p-RFP-*CYC1*t	This study
CF1173	IMX581 X-3::T1-*TDH3*p CORE-RFP-*CYC1*t	This study
CF1174	IMX581 X-3::T2-*TDH3*p CORE-RFP-*CYC1*t	This study
CF1175	IMX581 X-3::T3-*TDH3*p CORE-RFP-*CYC1*t	This study
CF1176	IMX581 X-3::T4-*TDH3*p CORE-RFP-*CYC1*t	This study
CF1177	IMX581 X-3::T5-*TDH3*p CORE-RFP-*CYC1*t	This study
CF1178	IMX581 X-3::T6-*TDH3*p CORE-RFP-*CYC1*t	This study
CF1179	IMX581 X-3::T7-*TDH3*p CORE-RFP-*CYC1*t	This study
CF1180	IMX581 X-3::T8-*TDH3*p CORE-RFP-*CYC1*t	This study
CF1181	IMX581 X-3::T9-*TDH3*p CORE-RFP-*CYC1*t	This study
CF1182	IMX581 Δ*hac1* T1-*TDH3*p CORE-RFP-*CYC1*t	This study
CF1183	IMX581 Δ*hac1* T2-*TDH3*p CORE-RFP-*CYC1*t	This study
CF1184	IMX581 Δ*hac1* T3-*TDH3*p CORE-RFP-*CYC1*t	This study
CF1185	IMX581 Δ*hac1* T4-*TDH3*p CORE-RFP-*CYC1*t	This study
CF1186	IMX581 Δ*hac1* T5-*TDH3*p CORE-RFP-*CYC1*t	This study
CF1187	IMX581 Δ*hac1* T6-*TDH3*p CORE-RFP-*CYC1*t	This study
CF1188	IMX581 Δ*hac1* T7-*TDH3*p CORE-RFP-*CYC1*t	This study
CF1189	IMX581 Δ*hac1* T8-*TDH3*p CORE-RFP-*CYC1*t	This study
CF1190	IMX581 Δ*hac1* T9-*TDH3*p CORE-RFP-*CYC1*t	This study
CF1191	IMX581 XII-5::*HAC1* X-3:: *TDH3*p-RFP-*CYC1*t	This study
CF1192	IMX581 X-3::R1-*TDH3*p CORE-RFP-*CYC1*t	This study
CF1193	IMX581 X-3::R2-*TDH3*p CORE-RFP-*CYC1*t	This study
CF1194	IMX581 X-3::R3-*TDH3*p CORE-RFP-*CYC1*t	This study
CF1195	IMX581 X-3::R4-*TDH3*p CORE-RFP-*CYC1*t	This study
CF1196	IMX581 X-3::R5-*TDH3*p CORE-RFP-*CYC1*t	This study
CF1197	IMX581 X-3::R6-*TDH3*p CORE-RFP-*CYC1*t	This study
CF1198	IMX581 X-3::R7-*TDH3*p CORE-RFP-*CYC1*t	This study
CF1199	IMX581 X-3::R8-*TDH3*p CORE-RFP-*CYC1*t	This study
CF1200	IMX581 X-3::R9-*TDH3*p CORE-RFP-*CYC1*t	This study
CF1201	IMX581 Δ*hac1* R1-*TDH3*p CORE-RFP-*CYC1*t	This study
CF1202	IMX581 Δ*hac1* R2-*TDH3*p CORE-RFP-*CYC1*t	This study
CF1203	IMX581 Δ*hac1* R3-*TDH3*p CORE-RFP-*CYC1*t	This study
CF1204	IMX581 Δ*hac1* R4-*TDH3*p CORE-RFP-*CYC1*t	This study
CF1205	IMX581 Δ*hac1* R5-*TDH3*p CORE-RFP-*CYC1*t	This study
CF1206	IMX581 Δ*hac1* R6-*TDH3*p CORE-RFP-*CYC1*t	This study
CF1207	IMX581 Δ*hac1* R7-*TDH3*p CORE-RFP-*CYC1*t	This study
CF1208	IMX581 Δ*hac1* R8-*TDH3*p CORE-RFP-*CYC1*t	This study

CF1209	IMX581 Δ*hac1* R9-*TDH3*p CORE-RFP-*CYC1*t	This study
CF1210	IMX581 X-3:: m94-*TDH3*p-RFP-*CYC1*t (0)	This study
CF1211	IMX581 X-3:: m94-*TDH3*p-RFP-*CYC1*t (-50)	This study
CF1212	IMX581 X-3:: m94-*TDH3*p-RFP-*CYC1*t (-100)	This study
CF1213	IMX581 X-3:: m94-*TDH3*p-RFP-*CYC1*t (-150)	This study
CF1214	IMX581 X-3:: m94-*TDH3*p-RFP-*CYC1*t (-200)	This study
CF1215	IMX581 X-3:: m94-*TDH3*p-RFP-*CYC1*t (-300)	This study
CF1216	IMX581 X-3:: m94-*TDH3*p-RFP-*CYC1*t (-400)	This study
CF1217	IMX581 X-3:: m94-*TDH3*p-RFP-*CYC1*t (-500)	This study
G1/Δ*hac1*	Δ*hac1* XII-2:: *TDH3*p CORE-*tHMG1*-*CYC1*t	This study
G2/Δ*hac1*	Δ*hac1* XII-2:: m94-m84-*TDH3*p CORE-*tHMG1*-*CYC1*t	This study
G3/Δ*hac1*	Δ*hac1* XII-2:: *TDH3*p-*tHMG1*-*CYC1*t	This study
G4/Δ*hac1*	Δ*hac1* XII-2:: m94-*TDH3*p-*tHMG1*-*CYC1*t (-50)	
G1/IMX581	IMX581 XII-2:: *TDH3*p CORE-*tHMG1*-*CYC1*t	This study
G2/IMX581	IMX581 XII-2:: m94-m84-*TDH3*p CORE-*tHMG1*-*CYC1*t	This study
G3/IMX581	IMX581 XII-2:: *TDH3*p-*tHMG1*-*CYC1*t	This study
G4/IMX581	IMX581 XII-2:: m94-*TDH3*p-*tHMG1*-*CYC1*t (-50)	
G1/*HAC1*	IMX581 XII-5::*HAC1* XII-2:: *TDH3*p CORE-*tHMG1*-*CYC1*t	This study
G2/*HAC1*	IMX581 XII-5::*HAC1* XII-2:: m94-m84-*TDH3*p CORE-*tHMG1*-*CYC1*t	This study
G3/*HAC1*	IMX581 XII-5::*HAC1* XII-2:: *TDH3*p-*tHMG1*-*CYC1*t	This study
G4/*HAC1*	IMX581 XII-5::*HAC1* XII-2:: m94-*TDH3*p-*tHMG1*-*CYC1*t (-50)	This study
MSBP003	*MATa ura3-52 tpi1 can1_::cas9-natNT2 TRP1 LEU2 HIS3*	This study (3)
F1	MSBP003/ pCP-*TPI1*p-Aamylase	This study
E1	F1 Kan-m94 *ERO1*p-*ERO1* / pCP-Aamylase	This study
P1	F1 Kan-m94-*PDI1*p-*PDI1* / pCP-Aamylase	This study
S1	F1 Kan-m94-*SEC24*p-*SEC24* / pCP-Aamylase	This study
BY4742	*MATa his3Δ1 leu2Δ0 lys2Δ0 ura3Δ0*	EUROSCARF
CF1120	BY4742 X3:: UPRE2m-*TDH3*p CORE-RFP-*CYC1*t	This study

1. D. Mumberg, R. Muller, M. Funk, Yeast Vectors for the Controlled Expression of Heterologous Proteins in Different Genetic Backgrounds. *Gene* **156**, 119-122 (1995).
2. Y. Zhang *et al.*, A gRNA-tRNA array for CRISPR-Cas9 based rapid multiplexed genome editing in Saccharomyces cerevisiae. *Nat Commun* **10**, 1053 (2019).
3. S. Xue *et al.*, Comprehensive Analysis of Signal Peptides in *Saccharomyces cerevisiae* Reveals Features for Efficient Secretion. *Advanced Science* **10**, 2203433 (2023).

Table S5 Primers used in this study.

Name	Sequence (5'→3')
**Plasmid construction**	
PEGP1	cgcctacttcttgcctattgtttcagtgcctttaaaccttGagctccagcttttgttc
PEGP2	tcgatatcaagcttGGATCCaattagttatgtcacgcttacattcac
EP1	AACAAAATGTCTGTTATCAAGCAAGTTATGAAAACCAAGTTACATTTGGAAGGTACCGT
EflinP2	CACCACCACCAGAACCACCACCACCGGAACCACCACCGCCAGGCAAAGCCTTTGGTAAT
EGP1	GTGGTTCCGGTGGTGGTGGTTCTGGTGGTGGTGGTTCTATGGTCTCCAAGGGTGAAGA
EGP2	ataactaattGGATCCaagcttgatatcgaattCTCACTTGTATAATTCGTCCATAC
GPDEP1	aaggtttaaaggcactgaaacaataggcaagaagtaggcgagtttatcattatcaatac
GPDEP2	TTCATAACTTGCTTGATAACAGACATTTTGTTatccgtcgaaactaagttctgg
TEFEP1	aaggtttaaaggcactgaaacaataggcaagaagtaggcggcacacaccatagcttcaa
TEFEP2	GTTTTCATAACTTGCTTGATAACAGACATTTTGTTttgtaattaaaacttagattaga
TPIEP1	aaggtttaaaggcactgaaacaataggcaagaagtaggcgagatctacgtatggtcatt
TPIEP2	TCATAACTTGCTTGATAACAGACATTTTGTTttttagtttatgtatgtgtttttgtag
CYCEP1	aaggtttaaaggcactgaaacaataggcaagaagtaggcgCCTCGAGCAGATCCGCCA
CYCEP2	TTCATAACTTGCTTGATAACAGACATTTTGTTTATTAATTTAGTGTGTGTATTTG
P2U P1	aattaaccctcactaaagggaacaaaagctggagctCTATGTCGACAAGCGGTTTACGT
P2U P2	ATTTTGAGGACACGTAATCCTTTTGTCGAGACACTTTTGACACGTAAACCGCTTGTCGA
PDCP1	CTCGACAAAAGGATTACGTGTCCTCAAAATCTCGAGTAGCTCATTTGAATCAGCTTATG
PDCP2	AGCCATGTTGTCTTCTTCACCCTTGGAAACCATTTTGTTTTTGATTGATTTGACTGTGT
PGKP1	CTCGACAAAAGGATTACGTGTCCTCAAAATCTCGAGaagggggtggtttagtttag
PGKP2	ATGTTGTCTTCTTCACCCTTGGAAACCATTTTGTTttgttttatatttgttgtaaaaag
TDH3P1	TCTCGACAAAAGGATTACGTGTCCTCAAAATCTCGAGCTAATAAGTATATAAAGACG
TDH3P2	GCCATGTTGTCTTCTTCACCCTTGGAAACCATTTTGTTTTTGTTTGTTTATGTGTG
TEF1P1	GTGTCTCGACAAAAGGATTACGTGTCCTCAAAATCTCGAGaataaaaatttttatcacg
TEF1P2	TAGCCATGTTGTCTTCTTCACCCTTGGAAACCATTTTGTTtttctttctaatgagcaag
TPI1P1	TGTCTCGACAAAAGGATTACGTGTCCTCAAAATCTCGAGtacctttggctcggctgctg
TPI1P2	ATAGCCATGTTGTCTTCTTCACCCTTGGAAACCATTTTGTTttttagtttatgtatgtg
P2U-RFP P1	AACAAAATGGTTTCCAAGGGTGAAGAAG
P2U-RFP P2	gtgacataactaattGGATCCaagcttgatatcgaattCTCATTTGTACAATTCGTCCA

PSNR52	GATCATTTATCTTTCACTGCGGAGAAG
gRNA-X-3	gcagtgaaagataaatgatcGACACATTAGTCTCGTATGTgttttagagctagaaatag
gRNA-XII-2	gcagtgaaagataaatgatcTTTGTCTTAGTCAAGCACGAgttttagagctagaaatag
gRNA-XII-5	gcagtgaaagataaatgatcCTATAACCGGTTTGAATTTAgttttagagctagaaatag
gRNA-hac1	gcagtgaaagataaatgatcATCGTACCGAGTGATGAACGgttttagagctagaaatag
PHMG P1	ATGGACCAATTGGTGAAGACTGAAG
PHMG P2	gtaatacgactcactatagggcgaattg
m94-GPD tHMG1 P1	AAGCGGTTTACGTGTCAAAAGTGTgctgaaaaaaaaggttgaaaccagtt
TDH3 P1	cctcactaaagggaacaaaagctggagctcagtttatcattatcaatactc
m94 TDH3 P2	tttttcagcACACTTTTGACACGTAAACCGCTTtttttccaaatcagagagagc
m94 TDH3 P3	AAGCGGTTTACGTGTCAAAAGTGTgctgaaaaaaaaggttgaaaccagtt
TDH3 P4	tggtgacttcagtcttcaccaattggtcCATTTTGTTTGTTTATGTGTGTT
m94-m84 TDH3 P1	ctcactaaagggaacaaaagctggagctcagctggagctctatgtcgac

Gene deletion, gene integration

X1	aaggtttaaaggcactgaaacaataggcaagaagtaggcgATACGGAGTACGTGTCAT
X2	tcgaattctttacttatattttcgctcttgagctcgtcctcaaattaaagccttcgagc
X3	aaggtttaaaggcactgaaacaataggcaagaagtaggcgNNNNSNNKYACGTGTCMNMMNWRNCTCGAGCTAATAAGTATATAAAG
Rhacp1	AGCACCCACCAGAACCTCTACAGCAGGCCTTCCAGGAGTGGTTAAGAGATGGAAATGAC
Rhacp2	AGCCAAGTTCGATTGCGAATTACTAGTTAGTTCAAAATCAGTCATTTCCATCTCTTAAC
Kgcn4 P1	CAATTTGTCTGCTCAAGAAAATAAATTAAATACAAATAAAgacatggaggcccagaata
Kgcn4 P2	GAGAATGAAATAAAAAATATAAAATAAAAGGTAAATGAAAcagtatagcgaccagcatt
Khaa1 P1	AAAGGAAACAAAAGTATAGAAAAAAAAAACCTAAAAAATAgacatggaggcccagaata
Khaa1 P2	ACTACAGTTACAGAGAAGCAAGAGACGAAAAGCAAATTTAcagtatagcgaccagcatt
XHP1	ATGAGTCACTGACAGCCACCGCAGAGGTTCTGACTCCTACTTCCTCTACTGGGCTTATC
XHP2	GAGTACGGCAGTTCGCTGTCACTGAACTAAAACAATAAGGGGTGTATTTAGGCTATGG
Hac1-E39A P2	CAGCTCTTCTGTTTCTCAAAATACGCTCGATCCTTCGCTGTGCCTTTTCCTCTTTTGTC
Hac1-E39A P3	AAAAGAGGAAAAGGCTCAGCGAAGGATCGAGCGTATTTTGAG
K60A P1	TTTGAGAAACAGAAGAGCTGCTCACCAGAGCAGAGAGAAAGCAAGACTACATCTGCAGT
mutant hacP2	TTCTCTCTGCTCTGGTGAGCAGCTCTTC
K60L P1	CAGAAGAGCTgctcaccagagcagagagaaattaagactacatctgcagtatc
K60G P1	AGAAGAGCTgctcaccagagcagagagaaaggaagactacatctgcagtATC

K60R P1	GAAGAGctgctcaccagagcagagagaaaagaagactacatctgCAGT
K60F P1	gaagagctgctcaccagagcagagagaaattcagactacatctgcagT
K60D P1	gaagagctgctcaccagagcagagagaaagatagactacatctgcAGT
K60C P1	gaagagctgctcaccagagcagagagaaatgcagactacatctgcagT
K60S P1	GAAGAGCTgctcaccagagcagagagaaaagcagactacatctgcagtat
K60W P1	gaagagctgctcaccagagcagagagaaatggagactacatctgcagtATC
K60E P1	GAAGagctgctcaccagagcagagagaaagagagactacatctgcAGTATC
K60Q P1	AAGagctgctcaccagagcagagagaaacagagactacatctgcAGTATC
K60P P1	AAGAGCTgctcaccagagcagagagaaaccaagactacatctgcagtatc
K60T P1	AGAGCTgctcaccagagcagagagaaaacgagactacatctgcagtat
K60M P1	AGAGCTgctcaccagagcagagagaaaatgagactacatctgcagtat
K60Y P1	AGagctgctcaccagagcagagagaaatatagactacatctgcAGTATC
K60V P1	AGAGCTgctcaccagagcagagagaaagtaagactacatctgcagtatc
K60N P1	AGAGCTgctcaccagagcagagagaaaaatagactacatctgCAGTATC
K60I P1	AGAGctgctcaccagagcagagagaaaataagactacatctgCAGTATC
K60H P1	AGagctgctcaccagagcagagagaaacatagactacatctgcAGTATC
RUP1	taggcaagaagtaggcgATACGGAGaACGTGTCtTAAAAACCTCGAGCTAATAAGTATA
RUP2	taggcaagaagtaggcgATACGGAcTACGTGTCAaAAAAACCTCGAGCTAATAAGTATA
RUP3	taggcaagaagtaggcgATACGGtGTACGTGTCATtAAAACCTCGAGCTAATAAGTATA
RUP4	taggcaagaagtaggcgATACGcAGTACGTGTCATAtAAACCTCGAGCTAATAAGTATA
RUP5	taggcaagaagtaggcgATACcGAGTACGTGTCATAAtAACCTCGAGCTAATAAGTATA
RUP6	taggcaagaagtaggcgATAgGGAGTACGTGTCATAAAtACCTCGAGCTAATAAGTATA
RUP7	taggcaagaagtaggcgATtCGGAGTACGTGTCATAAAAtCCTCGAGCTAATAAGTATA
RUP8	taggcaagaagtaggcgAaACGGAGTACGTGTCATAAAAAgCTCGAGCTAATAAGTATA
RUP9	taggcaagaagtaggcgtTACGGAGTACGTGTCATAAAAACCTCGAGCTAATAAGTATA
T1 P1	taggcaagaagtaggcgTACGGAGTACGTGTCATAAAAACCTCGAGCTAATAAGTATA
T2 P1	taggcaagaagtaggcgACGGAGTACGTGTCATAAAAACTCGAGCTAATAAGTATA
T3 P1	taggcaagaagtaggcgCGGAGTACGTGTCATAAAACTCGAGCTAATAAGTATA
T4 P1	taggcaagaagtaggcgGGAGTACGTGTCATAAACTCGAGCTAATAAGTATA
T5 P1	taggcaagaagtaggcgGAGTACGTGTCATAACTCGAGCTAATAAGTATA
T6 P1	taggcaagaagtaggcgAGTACGTGTCATACTCGAGCTAATAAGTATA
T7 P1	taggcaagaagtaggcgGTACGTGTCATCTCGAGCTAATAAGTATA
T8 P1	taggcaagaagtaggcgTACGTGTCACTCGAGCTAATAAGTATA

T9 P1	taggcaagaagtaggcgACGTGTCCTCGAGCTAATAAGTATA
dXP3	acgcgagggaagggaaataaggtttaaaggcactgaaacaataggcaagaagtaggcg
XII-5-Hac1 P1	ATGAGTCACTGACAGCCACCGCAGAGGTTCTGACTCCTACTT CCTCTACTGGGCTTATC
XII-5-Hac1 P2	GAGTACGGCAGTTCGCTGTCACTGAACTAAAACAATAAGGG GTGTATTTAGGCTATGG
XII-5-Hac1-238D P2	CCCTCTTGCGATTGTCTTCAATCAGTGATGAAGAAATCATTC
XII-5-Hac1-238D P3	TCATCACTGATTGAAGACAATCGCAAGAGGGTA
XII-5-Hac1-Med2 P6	TGATCCTGATCCAGAACCAGAACCTGAAGTGATGAAGAAATC ATTC
XII-5-Hac1-Med2 P7	ACTTCAGGTTCTGGTTCTGGATCAGGATCAATGGTAGTACAA AATAGC
XII-5-Hac1-Gal4 P1	ACTTCAGGTTCTGGTTCTGGATCAGGATCAGCCAATTTTAATC AAAGTGG
XII-5-Hac1-Gal4 P2	TCTTGCGATTGTCTTCACTCTTTTTTTGGGTTTGGTGG
XII-5-Hac1-Gal4 P3	CAAACCCAAAAAAAGAGTGAAGACAATCGCAAGAGGGTA
2UPRE2 P1	ataggcaagaagtaggcgATACGGAGTACGTGTCATAAAAACCTCGAC ATACGGAGTAC
2UPRE2P2	ACCGTCTTTATATACTTATTAGCTCGAGGTTTTTATGACACGTA CTCCGTATGTCGAG
m94 m94P1	ataggcaagaagtaggcgAAGCGGTTTACGTGTCAAAAGTGTCTCGAC AAGCGGTTTAC
m94 P2	ACCGTCTTTATATACTTATTAGCTCGAGACACTTTTGACACGT AAACCGCTTGTCGAG
m86 m86 P1	taggcaagaagtaggcgCAGTGGGTTACGTGTCATCAGAGCCTCGACC AGTGGGTTAC
m86 P2	ACCGTCTTTATATACTTATTAGCTCGAGGCTCTGATGACACGT AACCCACTGGTCGAG
m84 m84 P1	ataggcaagaagtaggcgAAAAGGATTACGTGTCCTCAAAATCTCGAC AAAAGGATTAC
m84 P2	ACCGTCTTTATATACTTATTAGCTCGAGATTTTGAGGACACGT AATCCTTTTGTCGAG
m94 m86 P1	ataggcaagaagtaggcgAAGCGGTTTACGTGTCAAAAGTGTCTCGAC CAGTGGGTTAC
m94 m84 P1	ataggcaagaagtaggcgAAGCGGTTTACGTGTCAAAAGTGTCTCGAC AAAAGGATTAC
UPRE2 m86 P1	ataggcaagaagtaggcgATACGGAGTACGTGTCATAAAAACCTCGAC CAGTGGGTTAC
UPRE2 m84 P1	ataggcaagaagtaggcgATACGGAGTACGTGTCATAAAAACCTCGAC AAAAGGATTAC
UPRE2 m94 P1	ataggcaagaagtaggcgATACGGAGTACGTGTCATAAAAACCTCG ACAAGCGGTTTAC
m94 UPRE2 P1	ataggcaagaagtaggcgAAGCGGTTTACGTGTCAAAAGTGT CTCGACATACGGAGTAC
I-KERO1 P1	GAATATAGTCATCCAGTAGCCATAGTTCACACACACATTAgacat ggaggcccagaata
I-KERO1 P2	cagtatagcgaccagcattc

I-KERO1 P3	catgcgtcaatcgtatgtgaatgctggtcgctatactgCTTATTCACCACATAAAGAAC
I-KERO1 P4	TAACGCCCAAAGCCACACTTTTGACACGTAAACCGCTTGGAAAAATGCGAAAAATTTCC
I-KERO1 P6	TTCTTTTTTGTTCTCTTCTTTTCTTTTTGTTCTTACTAACGCCCAAAGCCACACTTTTG
I-KPDI1 P1	ATTTGTTCGATGAAGTAAGCAGTTAATTCGTCCAGCTTCTgacatggaggcccagaata
I-KPDI1 P3	cgtcaatcgtatgtgaatgctggtcgctatactgCGGGGGTAACCTCGAAATCGTCGCA
I-KPDI1 P4	GCCCGACAGGGTAACACTTTTGACACGTAAACCGCTTACATATTATCACGTGCAGCCCA
I-KPDI1 P6	ACTAGTGAGAGAAAAGGGAAAAAGAGGCGCCGCCCGACAGGGTAACACTTTTGACACG
I-KSEC24 P1	TATACAACATCATGCTCTTAAAAGAATAGGTTACGTAATTgacatggaggcccagaata
I-KSEC24 P3	atgcgtcaatcgtatgtgaatgctggtcgctatactgAATTGATGGAAAATTTCAGC
I-KSEC24 P4	GAAAAAACAAGAAAACACTTTTGACACGTAAACCGCTTAAAAAAAAGAGAACAGCTAAG
I-KSEC24 P6	CGTTCTTCCAGTTTTCATTTTCTTCTGCCGTAAATGGAAAAAACAAGAAAACACTTTTG
X3-GPD P1	aaggtttaaaggcactgaaacaataggcaagaagtaggcgagtttatcattatcaatac
GU 0 P2	tacttattagACACTTTTGACACGTAAACCGCTTtcaagtaggggaataatttc
GU 0 P1	AAGCGGTTTACGTGTCAAAAGTGTctaataagtatataaagacg
GU 50 P2	tttcagcACACTTTTGACACGTAAACCGCTTtttttccaaatcagagagagc
GU 50 P1	AAGCGGTTTACGTGTCAAAAGTGTgctgaaaaaaaaggttgaaacc
GU 100 P2	taagaaaatgaACACTTTTGACACGTAAACCGCTTgatagatacatgcgtgggt
GU 100 P1	AAGCGGTTTACGTGTCAAAAGTGTtcattttcttacaccttctattac
GU 150 P2	tactccaggcACACTTTTGACACGTAAACCGCTTaggttgcatcactccattg
GU 150 P1	AAGCGGTTTACGTGTCAAAAGTGTgcctggagtaaatgatg
GU 200 P2	tgtttgtgccACACTTTTGACACGTAAACCGCTTcctgttctctgtagttgcg
GU 200 P1	AAGCGGTTTACGTGTCAAAAGTGTggcacaaacaggcaaaaaaacgg
GU 300 P2	atgccACACTTTTGACACGTAAACCGCTTagcttaaaaagcgggctcc
GU 300 P1	AAGCGGTTTACGTGTCAAAAGTGTggcatccagaaaaaaaaagaatcc
GU 400 P2	ACACTTTTGACACGTAAACCGCTTtttgggcatgtacgggttac
GU 400 P1	ccaaaAAGCGGTTTACGTGTCAAAAGTGTatagggggcgggttacacag
GU 500 P2	ttctttgaaaACACTTTTGACACGTAAACCGCTTtggcgagtattgataatgataaact
GU 500 P1	GGTTTACGTGTCAAAAGTGTtttcaaagaatacgtaaataattaatagtagtg
XII-2 tHMG P1	ACGATACTGCCAGATTTATGCAAAAAGAGAAAACCAAGGGgaacaaaagctggagctc
XII-2 tHMG P2	GCATCGGTGCTGTCCCTCCTTAGCTGAGGGTTCGGTGTACgtaatacgactcactatag
**Gene integration and promoter replacement verification**	
JX3P1	tttgatgtcggtcgtttgag
JX3P2	actcatatgaaatcttgctg

JHACP2	AGTCGACTTGAAGTTGGTAG
JHACP1	TGACAATTCAATTGATCTTG
JXII-5 P1	TTCGCCTGCTGCAAGATGAG
JXII-5 P2	ATAAGAATACTTCAGACGTG
RFPP3	GCCGAAGGTCGTCATTCCAC
RFPP2	CGTTGACGGAACCTTCCATGT
JGCN4 P1	TTGCTATCATGTACCCGTAG
JHAA1 P1	CAGGGAAGCTTTTCCAAGGA
JKERO P1	GAATACGTTCCTTTTTGTGC
JKPDI P1	TCGGCACACCACTTATAATG
JKSEC24 P1	TACTCTTAAACGTCCAAACC
KanaP2	catccatgttggaatttaatcg
JHMG P2	atccaagctttcaatatcgcg

Table S6 EMSA probes used in this study.

Probe	Sequence (5'→3')
UPRE2	gcgATACGGAGTACGTGTCATAAAAACctc
m82	gcgTTTACGGGTACGTGTCCTCAAAAGctc
m84	gcgAAAAGGATTACGTGTCCTCAAAATctc
m86	gcgCAGTGGGTTACGTGTCATCAGAGCctc
m94	gcgAAGCGGTTTACGTGTCAAAAGTGTctc
TDH3	gcgCCTTCTATTACCTTCTGCTCTCTCctc

The TDH3 probe was derived from a sequence located upstream of the core region of the *TDH3*p promoter.
